# Interaction between Purkinje Cells and Inhibitory Interneurons May Create Adjustable Output Waveforms to Generate Timed Cerebellar Output

**DOI:** 10.1371/journal.pone.0002770

**Published:** 2008-07-23

**Authors:** Simon Hong, Lance M. Optican

**Affiliations:** Laboratory of Sensorimotor Research, National Eye Institute, National Institutes of Health, Bethesda, Maryland, United States of America; University College London, United Kingdom

## Abstract

We develop a new model that explains how the cerebellum may generate the timing in classical delay eyeblink conditioning. Recent studies show that both Purkinje cells (PCs) and inhibitory interneurons (INs) have parallel signal processing streams with two time scales: an AMPA receptor-mediated fast process and a metabotropic glutamate receptor (mGluR)-mediated slow process. Moreover, one consistent finding is an increased excitability of PC dendrites (in Larsell's lobule HVI) in animals when they acquire the classical delay eyeblink conditioning naturally, in contrast to *in vitro* studies, where learning involves long-term depression (LTD). Our model proposes that the delayed response comes from the slow dynamics of mGluR-mediated IP3 activation, and the ensuing calcium concentration change, and not from LTP/LTD. The conditioned stimulus (tone), arriving on the parallel fibers, triggers this slow activation in INs and PC spines. These excitatory (from PC spines) and inhibitory (from INs) signals then interact at the PC dendrites to generate variable waveforms of PC activation. When the unconditioned stimulus (puff), arriving on the climbing fibers, is coupled frequently with this slow activation the waveform is amplified (due to an increased excitability) and leads to a timed pause in the PC population. The disinhibition of deep cerebellar nuclei by this timed pause causes the delayed conditioned response. This suggested PC-IN interaction emphasizes a richer role of the INs in learning and also conforms to the recent evidence that mGluR in the cerebellar cortex may participate in slow motor execution. We show that the suggested mechanism can endow the cerebellar cortex with the versatility to learn almost any temporal pattern, in addition to those that arise in classical conditioning.

## Introduction

**Figure 1:**
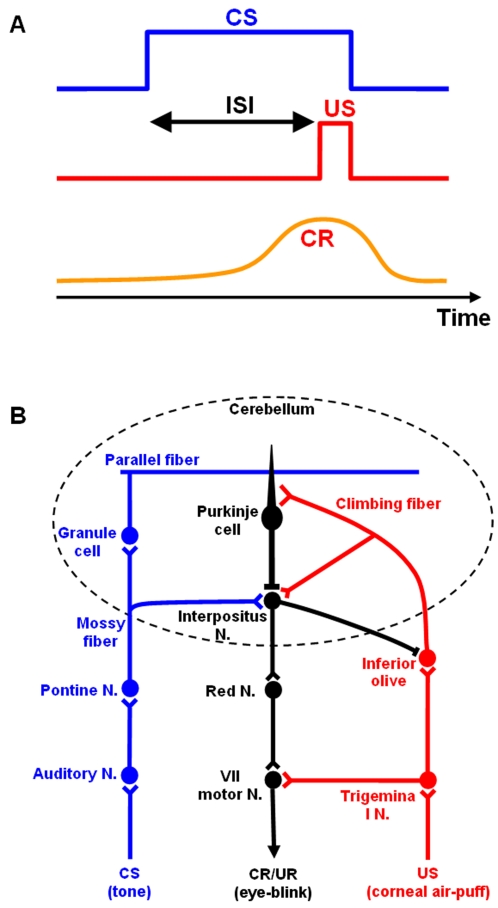
Classical delay eyeblink conditioning. A. conditioned stimulus (CS) and unconditioned stimulus (US) timing in delay conditioning. The inter-stimulus interval (ISI) is the time interval between the beginning of the CS and the beginning of the US. CS and US co-terminate. B. Pathways for classical delay eyeblink conditioning. The cerebellum receives CS and US signals during learning, resulting in an eye blink (the unconditioned response, UR). After learning, the cerebellum generates a delayed conditioned response (CR) to the CS that occurs just before the arrival of the expected US.

The cerebellum has an appealingly simple and orderly organization, and although its general function is still unknown, an enormous amount of information has been accumulated about its role in some simple movements over the past several decades. Key advances in our understanding come from animal studies of *classical delay eyeblink conditioning* [1], [2]. In this paradigm (see Figure 1A), the animal receives a conditioned stimulus (CS), such as a tone. After a certain delay (inter-stimulus interval, ISI), an unconditioned stimulus (US), such as an air puff directed at the cornea, causes a reflexive blink (unconditioned response, UR). The CS signal remains on until the delayed onset of the US signal, and the CS and US terminate at the same time. After training, the animal not only closes its eye in response to the tone but also learns to time (or delay) this conditioned response (CR) to achieve maximum eyelid closure when the US is expected [3], [4]. The cerebellum is necessary for this type of learning. For example, when its output (the superior cerebellar peduncle) is blocked, the expression of eyeblink conditioning disappears, but the animal's ability to learn the conditioning is unaffected [5]. The deep cerebellar nuclei (DCN), especially the anterior interpositus nucleus [6], [7], as well as the cerebellar cortex, Larsell's lobule HVI [8] and anterior lobe (Lobules I–V) [9], [10] are critical for learning this conditioning. Figure 1B summarizes the anatomical pathways known to support the classical delay conditioning. For more detailed discussion of these pathways, readers are referred to the earlier work by Thompson and his colleagues[2], [11].

The current explanation for the timing of the CR is summarized as follows [2]: The CS signal is transmitted to the Purkinje cells (PCs) via mossy fibers (MFs) and then parallel fibers (PFs), and the US is relayed to the PCs via climbing fibers (CFs). Conditioning somehow leads the PCs to disinhibit the DCN just before the beginning of the US. Activation of the DCN then drives the efferent pathway responsible for the expression of the CR. This cerebellar timing theory, in which the cortex controls the timed discharge of DCN activity, is consistent with cerebellar cortical lesion studies where maladaptively timed CRs occur after ablation [12] and pharmacological [13], [14] cerebellar cortical lesions. Although this cerebellar timing explanation is widely accepted, the mechanism for the appropriately timed responses of PCs and DCN neurons is not yet known.

Three mechanisms for learning the timing have been proposed: (1) the *Network state dependent* model [15], [16], [17]; (2) the *Spectral timing* model [18]; and (3) the *Adaptive-PC timing* model [19], [20]. The network state-dependent model postulates that spatiotemporally varying PF input patterns to PCs are used to learn the appropriate timing for the DCN disinhibition. In contrast, the other two models rely on differential responses of PCs to steady PF inputs, either because different PCs have different latencies of activation (spectral timing model), or because individual PCs can adaptively change their latency (adaptive-PC timing model). Although these models have been instrumental in shaping our understanding of cerebellar timing, they do not account for recent experimental findings. For example, one consistent finding is an increased excitability of PC dendrites (Larsell's lobule HVI) after acquisition of classical delay eyeblink conditioning[21], [22], [23]. In contrast, earlier models based on *in vitro* studies [24], [25] hypothesize a decrement or pause of PC activity for the timed generation of the CR. This contradiction between the findings in naturally conditioned animals and *in vitro* studies motivated us to seek a different theory of delayed conditioning.

In this study we propose a new model of cerebellar timing. The model emphasizes the interaction between the PC and its connected INs in generating modulated waveforms of PC activation. It is hypothesized that the delayed coupling of the CS and US delivered via PF and CF, respectively, induces a simultaneous increment of dendritic excitability in INs as well as in the associated PC, thus leading to an increased modulation of PC activity. This waveform modulation leads to a timed pause of the PC population and makes the DCN neurons generate the CR signals.

The learning-induced increment of dendritic excitability in the model derives from the observations that there are at least two time scales, called here short and long, evident in the cerebellar cortex. The short-time scale component is the glutamate-induced ionotropic component at the synapse between PF and the inhibitory neurons (i.e., stellate cells and basket cells) and at PF to PC synapses (PF→PC). This is the classical LTD/LTP mechanism. The long-time scale component is the metabotropic glutamate receptor (mGluR)-mediated long latency, long lasting component at PF→PC and at PF→IN synapses (see below). Whereas the ionotropic quick acting component has been assumed to mediate many varieties of motor learning, studies of the contribution of the mGluR-mediated slow component to motor execution are relatively recent [18], [26]. Convincing evidence of mGluR participation in motor execution comes from an mGluR-autoantibody study by Coesmans et al. [26]. They showed that: (1) acute application of human mGluR-autoantibody in mouse cerebellum blocked the mGluR on Purkinje cells and reduced PC neuronal excitability and firing rate; (2) when the autoantibody was introduced acutely to the mouse cerebellum, the performance of the vestibuloocular reflex dropped. Notably, the effect was more prominent for low frequency than for high frequency movements. These results show that the decrement of mGluR-mediated excitability in PCs may have affected the long-time scale component of motor execution.

We propose that INs also have a similar mGluR-mediated long-time scale component, thereby giving the IN-PC module full symmetry in temporal scales. Stellate cells bear both group I and group II mGluRs [27]. Moreover, recent results by Karakossian and Otis [28] indicate that the group I mGluRs at the PF→stellate excitatory synapse have similar signaling properties to those in the PC, and may have a similar time-delaying mechanism. (Interneurons also receive the Scheibel collaterals of CFs [29].) This gives the INs not only a fast acting, short-time scale capacity but also a slow acting, long-time scale component that could counteract or cooperate with the mGluR-mediated slow depolarization in PC dendrites.

Using these multiple-time scale mechanisms, our new model simulates the long-time scale calcium kinetics that is dependent on inositol 1, 4, 5-trisphosphate (IP3) via mGluR activation. The model can also replicate the observed LTP/LTD phenomena in the cerebellar cortex. Application of the model to delay eyeblink conditioning shows it learns timed conditioned responses. The model can also learn arbitrary temporal timing, as shown by its use to explain oscillations seen in a clinical oculomotor disorder [30].

## Results

**Figure 2:**
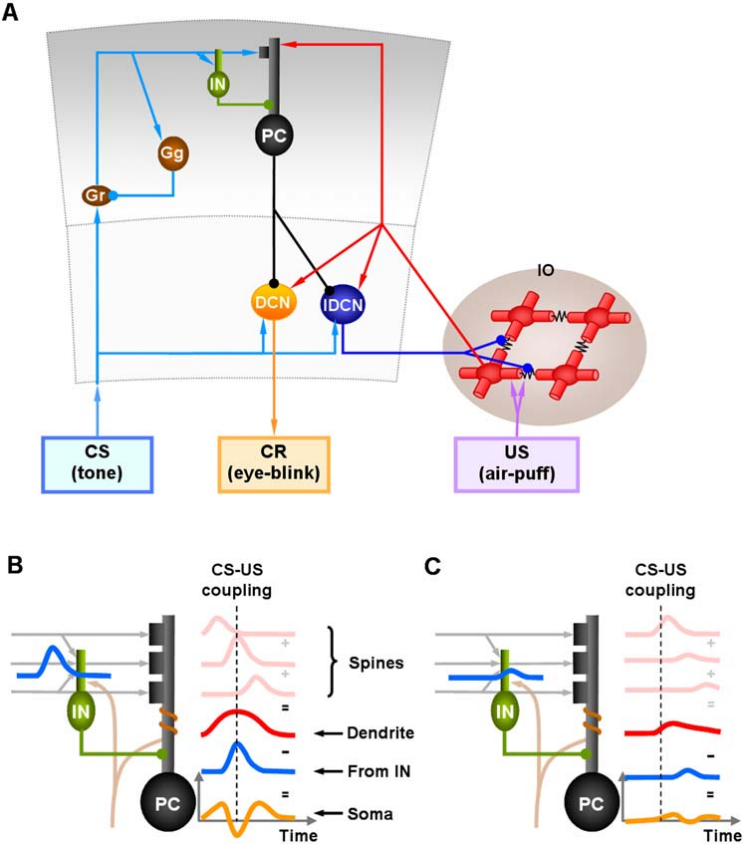
Schematic of model cerebellar cortex (CTX), deep cerebellar nucleus (DCN) and inferior olive (IO). A. Each module has two input pathways, a mossy fiber (MF in blue) and a climbing fiber (CF in red) pathway, and one output pathway from the deep cerebellar nucleus (DCN in orange). MFs project to granule cell-Golgi cell (Gr-Gg) networks (only one Gr and Gg cell are represented for clarity). At the intersection of the parallel fiber (PF) and CF pathways are the Purkinje cell-inhibitory interneuron (PC-IN) pairs. For simplicity the figure shows only one of the two IN for each PC. There are 54 PC-IN pairs in the simulation. IO contains noisy neurons that spike. The black zigzag lines between IO neurons (red) represent gap junctions. MFs carry conditioned stimulus (CS), and the climbing fibers carry an unconditioned stimulus (US), that should increase the output of the DCN (see text for details). Excitatory and inhibitory pathways are represented by triangular heads and round heads, respectively. B. Schematic of a PC-IN pair and its assumed signal processing. Each PC-IN module has one PC with two branches and two INs each inhibiting one PC branch. Each PC branch (PCBr) bears three spines where parallel fibers terminate. (Only one pair of IN-PCBr is shown for simplicity). The soma of the PC integrates the excitatory potential from the spines (red curve) and the inhibitory signal from the IN (blue) to generate the waveform of activation (orange curve). B and C. PC-IN pairs learn the timing of CS-US coactivation. When the CFs activate after the PF signal with a certain delay, the PC-IN pairs with a matching internal delay timing, such as the one in (B), increase their mGluR-mediated excitability. A learned increment of excitability in PC spines and the dendrite of IN after CF-PF coupling leads to smooth pulses of activation in PC spines (pink curves) and IN dendrites (blue curves) at PF input. (CFs contact INs via Scheibel collaterals of CFs [29]) The “CS-US coupling” with a vertical broken line in (B) and (C) shows the timing. After learning, the increased excitability leads to a big modulation of activity in the PCs of those pairs (the orange wave in B). This synchronized modulation of a population of PCs leads to the generation of timed CR. For the PC-IN pairs with different internal delay timings, such as the long delay timing in (C), the CS-US coactivation does not change their excitability. Theses pairs remain relatively inactive therefore do not contribute to CR.

This computational study constructs a model of the cerebellar circuit utilizing leaky integrator-type equations for all of the cell types except the inferior (IO) olive neurons. IO neurons use more elaborate spiking equations to simulate realistic CF activity including the low frequency (∼2 Hz) baseline noisy spikes[31]. (NB: In all the simulations every component of the model runs simultaneously, including the noisy IO neurons.) Figure 2A illustrates the simulated circuit of the cerebellum. There are four major divisions in the circuit: Two inputs (MF and CF), the cerebellar cortex, and the deep cerebellar nucleus (DCN). MFs provide inputs that represent certain events (CS in Figure 2A), such as a tone signal in the classical eyeblink conditioning paradigm[6], [32], [33], [34]. This input is transmitted to the cerebellar cortex via granule cells and also to the DCN by MF collaterals. CFs, which constitute another major input system, originate in the inferior olive (IO) and transmit US signals [35]. The DCN activity represents the output of the cerebellum (causing the CR in Figure 2A)[11]. The DCN also gates the IO-mediated learning in the cerebellar cortex with its negative feedback via inhibitory DCN projections (IDCN→IO)[36]. This makes the DCN an integral part of the learning circuit of the cerebellum.

In our model, the timing mechanism is localized to the PC-IN pair. Figure 2B summarizes the key concepts of signal processing in a PC-IN pair. First, it is assumed that repeated CF-PF coupling trains the PC spines and the IN dendrite to increase their excitability. After this training, the increased excitability makes the spines of the PC and the dendrite of the IN increase their potentials upon PF input due to the mGluR-induced long latency calcium activation (the terms *increment* or *decrement of excitability* will be used here for long-time scale plastic changes to differentiate them from the well known LTP and LTD phenomena, which historically refers to the changes in the fast AMPAR pathways[37]). It is assumed that whereas the IN shows a narrow activation profile (blue trace in Figure 2B), the PC dendrite generates a relatively broad activation profile (red trace) because of the variability in latencies of the slow-acting intracellular calcium components among the dendrite's many spines (three pink traces). At the PC dendrite level, the excitatory potential coming from the PC spines (red trace) and the inhibitory potential coming from the IN (blue trace) interact. This interaction generates a waveform, or temporally modulated pattern, of potential in the PC soma (orange trace in Figure 2B). We assume that PC-IN pairs have a wide range of mGluR-induced activation latencies that span the possible range of delay timing, similar to the range of PC latencies in the population-based spectral timing model [18]. The modulation of the waveforms of PC activations via the PF-CF coactivation happens only in those PC-IN pairs whose latencies match the timing of the coactivation. For example, a PC-IN pair whose internal timing longer than the CS-US coupling timing, as illustrated in Figure 2C, will not increase the excitabilities, and the PC's activation will not be modulated. This way only the PC-IN pairs having the right internal timing will be recruited by the CS-US coupling leading to a timed decrease of discharge in the PC population. This decrement of inhibition lets the DCN generate the conditioned response.

In the following section, we first show that the model can generate some known properties of the PC by simulating the calcium activation profiles in the PC spine following PF and CF signals. Next we show that the model is able to replicate the well known LTD/LTP data in PF→PC synapses with its learning rules. An extension of this learning rule at PF→IN synapses is also simulated based on a recent observation by Rancillac and Crepel [38]. Then we show the learning properties of the long-time scale component using examples of the delay conditioning paradigm. This example will show how the model uses the long latency calcium components in PCs and INs to learn to generate appropriate temporal waveforms in the PCs, which then gives the PC population a timed pause. As further examples, CRs with 3 different delay timings and double-response learning will be simulated as evidence of the model's flexibility. Finally, a simulated IO lesion is performed to show that the model's overall network-wide behavior matches the reported *in vivo* results after IO lesions.

### Simulation of signaling properties in PC spine

**Figure 3:**
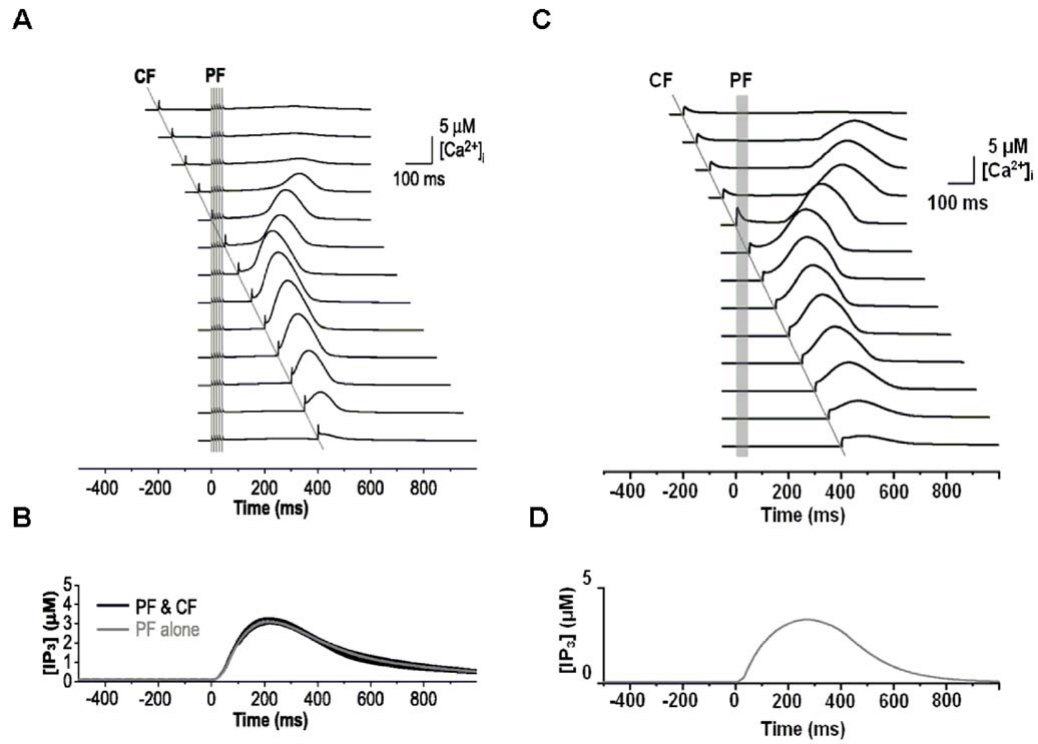
Slow activating regenerative Ca^2+^ release dependence on the timing of PF-CF inputs. A. Simulation results by Doi et al. [39] on Ca^2+^ activations at conjunctive PF-CF inputs with various intervals between the PF input and CF input. The PF input is delivered at 0 ms, indicated by the five vertical lines in panel A and a vertical gray bar in panel C. CF signal is given at −200∼+400 ms, as indicated by the single diagonal line. B. IP3 time courses in response to the various PF-CF inputs. Note that the IP3 profiles are almost identical regardless of the timing of PF-CF inputs or even for PF input alone. C. Our new model generates profiles of calcium activation similar to the model in A. D. Simulation by our model of IP3 activation at PF input. The new model adopts the results in panel B and uses just one IP3 activation profile (similar to *PF alone* in panel B) in generating the various Ca^2+^ profiles at various timing of PF-CF inputs in C. Panels A and B are from Doi et al. [39], with permission, copyright (2005) by the Society for Neuroscience.

In their theoretical study, Doi et al. [39] simulated the intracellular mechanism of PF- and CF-induced calcium concentration change in the PC spine. They showed that the regenerative intracellular calcium activation upon PF input depended on IP3 activation via mGluR stimulation (Figure 3A). One interesting finding was that the profile of IP3 activation during PF-CF coupling did not depend on anything except the PF signal-induced mGluR activation (Figure 3B). Using the simplified mechanism of IP3 activation (one profile shown in Figure 3D), our model was able to simulate calcium activation profiles depending on PF-CF signal timing (Figure 3C). Both models generate similar PF-induced IP3 activation profiles and long-time scale calcium activation profiles. This demonstrates that despite its simplified mechanism, the current model's PC spine replicates the suggested PF and CF signal-induced intracellular calcium changes.

### LTP/LTD in PF→PC and PF→IN synapses

**Figure 4:**
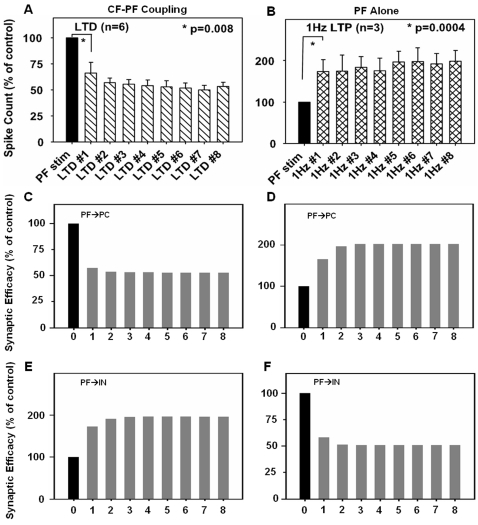
Simulation of changes in synaptic efficacy. A. Induction of LTD from Lev-Ram et al. [40]. Eight rounds of co-stimulation of CF and PF at 1 Hz for 30 seconds caused depression at PF→PC synapse leading to a decrease in spike count (an indirect measure of synaptic efficacy). B. Induction of LTP from Lev-Ram et al. [40]. Stimulation of PF alone induced an increase in spike count (note change of scale). C. Co-stimulation of PF and CF induces LTD in PF→PC synapse, similar to the change in spike count in A. D. Stimulation of PF alone induces LTP in PF→PC synapse, similar to results in B. E. PF-CF co-stimulation induces LTP at PF→IN synapse. F. Stimulating PF alone induces LTD at PF→IN synapses. Note that the same input causes adaptive changes in opposite directions at PF→PC and PF→IN synapses. Data in panels A and B are reproduced with permission from Lev-Ram et al. [40], copyright (2003) National Academy of Sciences, U.S.A.

Figure 4 shows the experimental data (Figure 4A and B) by Lev-Ram et al. [40] and the simulation results of the current model (Figure 4C–F). The experimental data show that coactivation of PF and CF signals induces depression in PF→PC synapses (Figure 4A). The model simulates the findings showing a similar amount of depression (∼50% of control) in PF→PC synaptic strength at PF-CF co-stimulations (Figure 4C). The result of the simulation also shows that the plasticity in the PF→IN synapse has the opposite direction of change (Figure 4E). Figure 4B shows an LTP paradigm where PF stimulation alone induces an increment of synaptic strength at PF→PC. The model simulates this increment of synaptic efficacy (∼200% of control) at PF→PC when the PF occurs alone (Figure 4D). The direction of plasticity at PF→IN synapse is the opposite (Figure 4F), consistent with the observation by Rancillac and Crepel [38].

**Figure 5:**
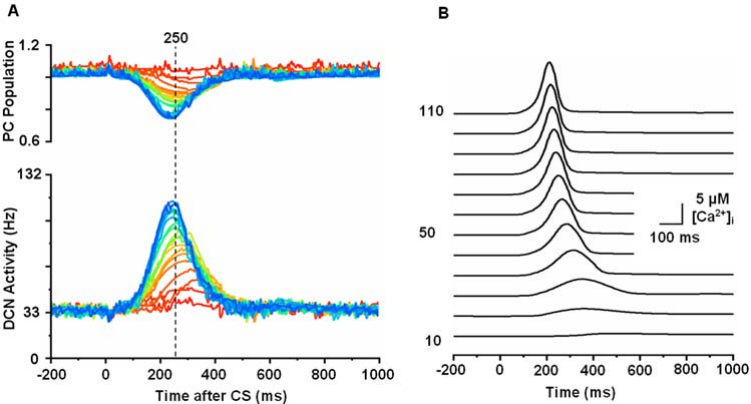
Simulation of learning in PC population and DCN neuron. A. Upper panel: Traces of postsynaptic weighted sum in DCN of outputs from the PC population. Lower panel: Traces of DCN neuron activity with a baseline firing rate of about 33 Hz. Twenty test trial responses (every 15th trial during learning) are shown, starting at the beginning of training (red) and progressing to the learned state after 300 trials (blue). The ordinate of the upper panel has an arbitrary scale. The number (250) above the broken line indicates the ISI time in ms. Subsequent figures use these same conventions. B. Increment of excitability and early onset of the peak of PF-induced [Ca^2+^]*_i_* profile. The curves show PF-induced [Ca^2+^]*_i_* profiles in the model PC spine at PF-alone test trials after different numbers of PF-CF conjunctive trials (indicated on the left side of the figure, 10∼110 times). Every 10^th^ trial is shown for clarity. The curves show that as the PF-CF conjunction progresses, the model spine responds with an increased volume of [Ca^2+^]*_i_* at earlier times due to the facilitated kinetics of calcium activation. The time shift of the peak saturates as the learning progresses. See text for more details.

**Figure 6:**
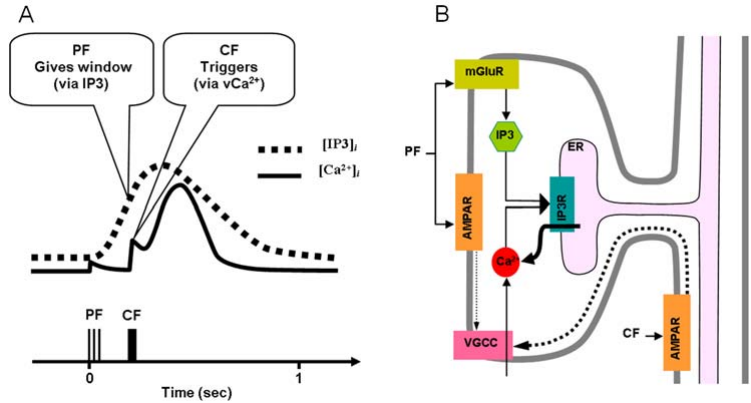
The simplified scheme for slow-rising calcium activation used in the simulation. A. Illustration of conclusion by Doi et al.[39]. PF generates IP3 activation profile (dotted curve), which gives the same window for regenerative Ca^2+^ activation irrespective of CF signal. CF signal triggers the regenerative Ca^2+^ activation (the round big hump, later part of solid trace) by mobilizing voltage-gated calcium (vCa^2+^) influx via voltage-gated calcium channels (VGCCs). The lower part of the figure shows the PF, CF signals. B. The circuit used by the current model. The figure shows signal pathways recruited by PF and CF. The big arrowhead with two stems indicates a multiplicative process that realizes the cooperative actions by IP3 and vCa^2+^ explained in panel A. Only the excitatory interactions (arrows) are shown for clarity. The thick black arrow indicates the calcium release from the endoplasmic reticulum (ER). See text for details. IP3R: IP3 receptor.

Figure 5A shows the progress of learning in delay eyeblink conditioning. The upper panel of Figure 5A shows the weighted sum of PC population activity (i.e., the input to the simulated DCN neuron). Each trace is recorded at every 15^th^ test trial when only the PF signal is provided. As the learning continues, the trough of the PC population signal deepens and shifts to an earlier time. The peak of the DCN activity shown in the lower panel of Figure 5A reflects these changes and becomes bigger and earlier as the learning progresses. The peaks shift because of the change in the intrinsic property of the calcium signaling kinetics due to the learned increased excitability of the pathway (PF→PC, PF→IN). Figure 5B shows an example where PF-CF coupling changes the peak latency and amplitude of the calcium activation profile. The result for every 10^th^ trial is shown for clarity. The shift is due to the facilitated calcium positive feedback mechanism that makes the calcium influx from the endoplasmic reticulum (ER) faster (Figure 6). This peak shift contributes to the shift of response profiles in the PC population and DCN as shown in Figure 5A. One thing to note is that the shift saturates (Figure 5B) as learning continues because of the limited time window given by the IP3 activation profile (Figure 6).

To simulate the slow time scale calcium kinetics the model adopts the theoretical findings by Doi et al. [39]. They showed that a PF signal generates an IP3 activation profile that acts as a window in which the CF signal can trigger the slow-rising calcium component (Figure 6A). Figure 6B shows the circuit in our model that simulates the process of the regenerative calcium release (the thick arrow) from the ER (Eq. 7). The model shows that the PF signal stimulates mGluRs, which in turn activates IP3. When this IP3 activation is combined with an elevated level of [Ca^2+^]*_i_* caused by CF→AMPAR activation of the voltage-gated calcium channel (VGCC), it causes positive calcium feedback by releasing calcium ions from the ER. The big arrowhead with two stems in Figure 6B indicates this multiplicative process. Our model also includes a similar slow-acting calcium activation mechanism in the IN dendrites, because of the recent finding by Karakossian and Otis [28] that both PCs and INs have a slow-acting long-time scale excitatory component mediated by an mGluR pathway (see Methods for more details).

**Figure 7:**
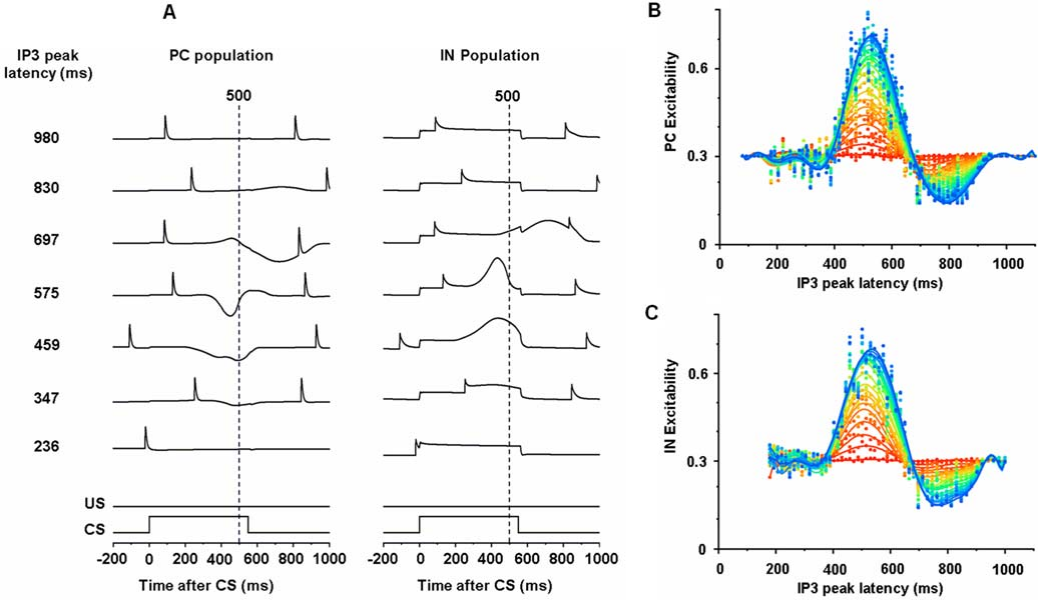
Learned increased excitability of PCs and INs. A. Each row shows responses of a pair of a PC and an attached IN to the CS during a test trial (no US is given; see US, and CS signals at the bottom of each column). Every eighth PC-IN pair among 54 modules is displayed. Learning was induced by CF-PF couplings with an ISI of 500 ms. While PCs and INs show responses to the PF input, the modulations are noticeable only in the pairs having IP3 peak latencies (left column) around 500 ms (three middle traces). The narrow peaks are CF-induced activations. The broken lines indicate the time when the US is delivered (500 ms after the CS) in pairing trials. Panels B and C show the concurrent development of learned long-time scale excitabilities (the dots) in PC and IN subpopulations (with IP3 peak latencies ∼500 ms) that are responsible for the PC-IN interactions shown in A. Every 25^th^ trial is shown for clarity (red to blue is early to late). The curves in corresponding colors are polynomial fits in each learning stage. Also note the troughs (around 800 ms) after peaks (see text for explanations).

**Figure 8:**
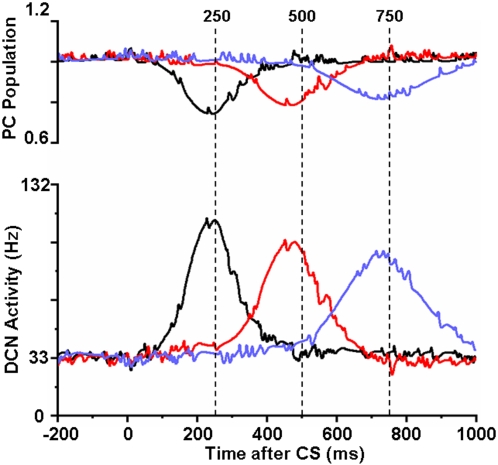
Simulation of delay eyeblink conditioning. Upper panel: Input given to the simulated DCN neuron from the PC population with different ISIs (black curve: 250 ms, red: 500 ms, blue: 750 ms). Lower panel: DCN activities at different ISIs. PC population reduces the inhibitory output near the arrival of the air-puff US signals (dashed lines). The model DCN peaks near the arrival of the US with appropriate delays because of the reduction of PC inhibition. The numbers above the broken lines (250, 500 and 750) indicate the ISI times in ms. Note that the above profiles simulate the timed CR generated by the cerebellum (e.g. Figure 2. in [51], and Figure 14B in [104]) without the influence of the cerebral cortex. Decerebrate animals show timed eye blink signals without anticipatory eyelid movements, which are seen in animals with cerebrum (e.g. [105]).

Figure 7A shows seven PC-IN pairs with different IP3 peak latencies. Repeated timed couplings of CS-US (ISI 500 ms) have trained the PC-IN pairs with similar IP3 peak latencies (the 3 rows around the middle with IP3 peak latencies of 459, 575 and 697) to modulate their calcium kinetics to decrease the PC activation profile. One example of the concurrent increase of excitabilities in PCs and INs is shown in Figure 7B and C. As the learning progresses (from red to blue in the figure), the PCs and INs with the IP3 peak latencies around 500 ms gradually increase their excitabilities (the dots in the figure). The curves with corresponding colors are the polynomial fits of the excitabilities across the population in each learning stage. The figure also shows that the PCs and INs that have a bit longer latencies become depressed (e.g., troughs around 800 ms) as a result of learning. This depression reflects the post-firing-refractory period of IO observed in the experiments (e.g., [41], [42]; unpublished findings from recordings in the medial accessory olive from our laboratory). The decreased excitabilities in PCs and INs do not affect the performance of the learned generation of the CR, because the depressed PCs and INs simply do not generate the long-time scale potentials at the CS, and are thus irrelevant to the expression of the CR. Figure 7A shows PCs having a variety of responses, often with multiple peaks similar to the patterns *in vivo* [43]. However, their population response shows a smooth trough around the arrival time of the US (e.g. Figure 8). PC-IN pairs that have IP3 peak latencies far from the given ISI usually react with an insignificant modulation of their activity. The sharp spikes are CF signal-induced PC activations. Figure 8 shows simulations of the model with ISIs of 250 ms, 500 ms and 750 ms. The troughs of the PC population and the peaks of the DCN neuron occur near the arrival time of the US (dashed lines).

**Figure 9:**
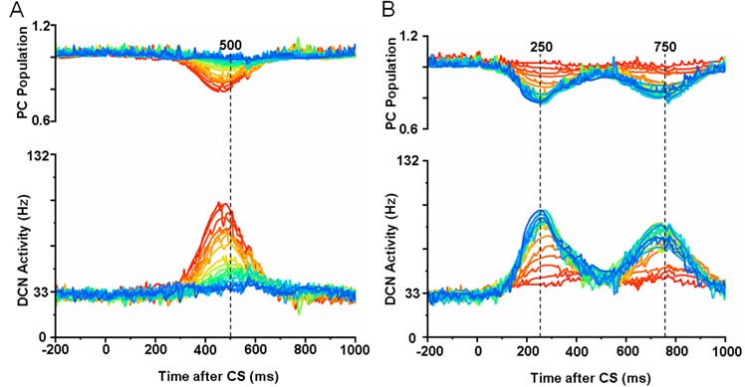
Extinction and double response. A. The progress of simulated extinction in DCN activity and the weighted sum of PC population activity are shown. Every 25^th^ trial is shown for clarity (red to blue is early to late). At the end of the training with ISI 500 ms (the first trace here, in red, is the same as the red curve in Figure 8) the CS signal was presented alone, without a US signal, causing extinction. B. Response to double-puff unconditioned stimuli. Progress of training with two alternating ISIs (US at 250 ms or at 750 ms) after one CS is shown. Every 30^th^ trial of 600 trials is shown (red to blue is early to late).

The model shows extinction when it receives CS-only trials. Figure 9A shows an example of extinction after acquisition with 500 ms ISI (color indicates order of traces; red is early, blue is late). Extinction took a course similar to acquisition, but in reverse (cf. Figure 5A).

### Double response

If the animal is trained with two different ISIs interleaved randomly, the animal learns to respond to the CS with two eye blinks around the time of the two ISIs [44], [45]. To simulate this experimental data, the model was trained with alternating trials where the CS was coupled with one US at a short ISI of 250 ms or one US at a long ISI of 750 ms. Figure 9B shows the result of the simulation. The model learns to produce two peaks, one right before the expected arrival of each of the two USs. To induce the double response, the strength of the US signal was increased to 140% of the control strength used for all the rest of the simulations. The need for an increased US strength for the induction of a double response has been well documented [45].

### IO lesion and its long-term effect in cerebellum

We examined the long-term effect of an IO lesion on the cerebellar circuit. To simulate the model for such a long time (over one thousand days), a lumped model was used (see: Supplementary Information, Note 3 in Text S1). This simplification was possible because of the absence of the IO and the baseline activity-only MF inputs to the cerebellum, which eliminated any learning activity in the network. See the Supporting Information, Note 3 in Text S1 for the correspondence of the lumped model to the original cerebellum model (Supplementary Figure S3) and a detailed explanation of it. The pre-lesion behavior of the cerebellar modules including the IO and DCN are also shown in the Figure S2 in the Supporting Information for comparison (see also Supplementary Note 2 in Text S1).

**Figure 10:**
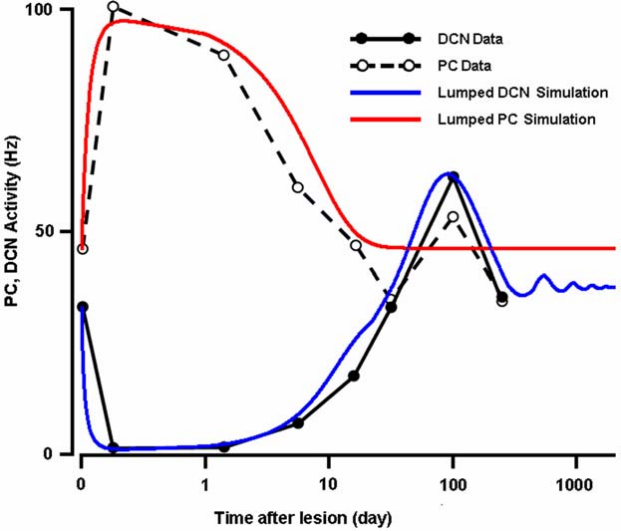
Simulation of post-IO lesion activities of PC and DCN. The experimental data of DCN (filled dots and black line segments) and PC (blank dots and dashed line segments) firing rates are from Batini et al. (1985). The model predicts beyond experimental data points (250th day) that, (1) the PC (in red) recover their baseline firing activity to the pre-lesion level and, (2) the DCN activity (in blue) stabilizes at an elevated level. These two predictions are consistent with the observation by Billard and Daniel [47]. See the Supporting Information Note 3 in Text S1 for detailed mechanisms of the network behavior.

Figure 10 shows the lesion data and the simulation results. The black lines indicate the average baseline activities of PCs (open dots and dashed lines) and DCN neurons (filled dots, solid lines) observed after IO lesion [46]. The red and blue curves in Figure 10 are simulation results indicating baseline PC and DCN activities, respectively. The experimental data show that the IO lesion immediately induces an increment of firing rate in the PCs, followed by a very slow decay back to their pre-lesion level. The simulation results show the same trend, but the red trace (PC) misses the small fluctuation (<25% of the pre-lesion level) in the data between 30–100 days, indicating a more elaborate mechanism may be needed to explain this small fluctuation. The simulated recovery of the PC firing rate to its pre-lesion level (∼50 Hz) is consistent with the observation by Billard and Daniel [47], who found a stabilized pre-lesion level PC activity two years after CF deafferentation. The DCN neurons are the recipient of PC output and become depressed after an IO lesion because of the increased inhibition from the PCs. As the PCs slowly recover to their pre-lesion baseline firing rate, DCN neurons also recover their activity to their pre-lesion level. However, the increment of their firing rate passes their pre-lesion level and increases to a level double (∼200%) the pre-lesion level. Data from Batini et al. [46] and Billard and Daniel [47] indicate that after the initial post-depression peak (the peak in the black dashed curve at around 100 days in Figure 10) the DCN activity stabilizes at a higher level than it had before the lesion. The model shows the same result, with a damped oscillatory behavior of DCN neuron activity after about 700 days (wiggly end of blue trace in Figure 10). A more parametric explanation of the behavior of the network is given in the Supporting Information Note 3 in Text S1.

## Discussion

The model described in this article simulates cerebellar timing in classical delay eyeblink conditioning. Generalizing the observation that acute blockage of the mGluR-mediated intracellular mechanism disturbs long-time scale motor execution[26], we hypothesize that the long-latency calcium activations in PC spines and IN dendrites modulate the activity of the PC so that it pauses at the correct time. This temporally-modulated waveform, which constitutes the output of the cerebellar cortex, is assumed to be modifiable by repeated coupling of PF and CF signals. The following discussion considers the assumptions and limitations of the model.

### Timing in Purkinje cell-inhibitory neuron module

The current model hypothesizes that (1) the interplay between IN and PC generates the PC pause and (2) that the slow-activating mGluR-mediated [Ca^2+^]*_i_* change in PC [48], [49] and in IN [28] determine the timing of the pause. There are three main reasons to propose these two hypotheses. First, mGluR may be important for long time-scale motor execution. This is supported by a recent experiment by Coesmans et al. [26] where they showed that an acute blockage of mGluR activation by application of mGluR1-autoantibodies in the cerebellar cortex impaired the motor performance more for lower frequency than for higher frequency motor execution. Second, the electrical stimulation experiment by Shinkman et al. [50] suggests that the timing mechanism is somewhere after the PF, because PFs may not carry time-varying information (see also [51]). Therefore the timing may be generated either by the PC alone or by the interplay between the PC and associated INs. Third, a consistent experimental finding is that a conditioning-specific change happens in the PC dendrites in the form of an increment of excitability [21], [22], [23]. This creates a paradox: if learning increases PC excitability, how can they pause at the right time? Our model suggests a solution to this paradox by proposing a mechanism that generates a simultaneous increment of activity in PC and IN as a consequence of learning. This concept is a temporal version of the shaping of spatial waveforms in the visual system. For example, in V1 excitatory and inhibitory neurons both increase their activation in response to a relevant input, which shapes the spatial waveforms that define the (center-surround) receptive fields of visual neurons [52]. Similar to the properties of the spatial center-surround “filter”, which is known to be able to encode almost any visual inputs, the temporal filter suggested in this study requires similar center and surround temporal envelops to encode a given temporal pattern. However, a certain exact configuration, such as the one used in this study (a main one and two others at −100 ms and +100 ms), is not required (result not shown). As in the center-surround spatial filter, it is the combined shape of the center-surround filter, not the specific underlying latencies, that enables the temporal encoding ability. We speculate that the modulation of both PCs and INs endow the cerebellar cortex with the versatility to learn almost any temporal output pattern (e.g., we have modified our model to reproduce oculopalatal tremor, a clinical eye movement disorder [30]).

### Contribution of short-time scale component in cerebellar timing

The current study examined the possible roles of long and short time scales in cerebellar timing by constructing a model cerebellum with a fairly complete circuit (with MF, CF, Gr-Gg, PC-IN, DCN and IO modules) and implementing the two signaling pathways (AMPAR, mGluR pathways) in the PC-IN pairs. Unlike our initial expectation, which envisioned some weighted contributions from both scales, we did not see any significant contribution of the short time scale in learned cerebellar timing (Note 1 in Text S1 and Figure S1 in the Supporting Information). There are two factors in the model that lead to this conclusion: First, the CS-US coupling in the delay conditioning did not systematically induce LTP/LTD at the PF→PC synapse. This is because of the way the CS and US interact in the model as in Eq. (8). As described above, LTD occurs at the PF→PC synapse when CS and US is coupled due to the surge of [Ca^2+^]*_i_*, and LTP happens when CS is given alone. In classical delay conditioning, however, the CS-US coupling happens with a long CS alone period (∼250 ms up to 4 s) before the US unlike *in vitro* LTD paradigms where there is little, if any, delay between the PF stimulation and the CF stimulation (e.g. [40]). Because of this relatively long delay between the start of the CS and the beginning of the US, the LTP (during the CS alone period) and LTD (CS-US coupling period) coexist in the model, therefore driving the synaptic weight to a stable attraction point defined by the coefficients of Eq. 8 (see below). It needs to be emphasized that most *in vitro* LTP/LTD experiments are done in a way incompatible with *in vivo* classical delay condition. For example, inter-stimulation period is very short (e.g. 1 s, [24]) in *in vitro* studies compared to natural classical delay conditioning (intertrial interval (ITI)>5 s; longer ITI is more effective). Considering the fact that the CS-induced mGluR effect may last more than a second, the short inter-stimulation period the *in vitro* LTP/LTD experiments does not seem to reflect the situation of the classical delay conditioning. Second, the model adopts a shunting mechanism whereby PC population activity gates the MF input to DCN. This mechanism allows the modulation of DCN activity by the MF signal only when the PC population decreases its activity, thus eliminating the need for an immediate increase of PC population activity to suppress the early component of DCN activity during the ISI period. This hypothesis is consistent with recent findings by Aksenov et al. [53], who showed that the firing rate of the DCN neurons does not show early activity during the ISI period, even after pharmacological manipulations (application of GABA_A_ antagonist, agonist and AMPAR, mGluR antagonist) of the related cerebellar cortex. It is, however, difficult to rule out completely the possible need for an elevated cortical inhibition during the ISI period. This is due to the intricate level of inhibition required for the gating of the MF→DCN pathway [54]. An alternative possibility is that another part of cerebellar cortex, such as the anterior lobe, may have a permissive control for expression of the conditioned response. This view is consistent with lesion studies where it was found that the anterior lobe was needed for extinction of the conditioned response [9], and that the PCs tended to increase their firing rate during the early part of the CS signaling and decrease during later portion of the CS signaling [10]. Further experimental and modeling studies are needed to clarify this issue.

### Deep cerebellar nucleus module

The current model implements the presumed plasticity at MF→DCN synapses, as have earlier models [15], [17]. This assumed plasticity is consistent with the early work by Miles and Lisberger [55], who predicted the existence of plasticity in the vestibular nucleus (which is the output target of the vestibular cerebellar cortex) as well as in the cerebellar cortex (see [56], for more references). In the case of delayed eyeblink conditioning, the level of learning was related to the formation of excitatory synapses on DCN neurons [57]. Consistent with this observation, Chen and Steinmetz [58] also found that activation of protein kinase in DCN is needed for acquisition but not expression of delayed conditioning. The model hypothesizes CF driven learning mechanism at this MF→DCN synapse. However, the exact learning mechanism at this synapse needs further investigation.

Currently, there is no data showing how much the MF→DCN pathway contributes to motor execution, nor is there any data about the contribution of the cortical part to motor execution. Medina et al. [17] showed in their experimental and modeling work that a partial lesion of the cerebellar cortex leads to an early increase of DCN activity during the ISI period, which goes away with more training. They concluded that the MF→DCN pathway provides the “fuel” for DCN activity, and the cortical input modulates that input to express a timely increase of the DCN firing rate. However, DCN neurons also show a rebound potential after hyperpolarization [59], [60], which may contribute to the firing rate during motor execution [61]. Our current model takes a middle ground by assuming that the discharge of the PC population can induce DCN firing by inducing rebound depolarization, in addition to its gating role on the input from MF collaterals.

### Inferior olive module

Numerous studies have suggested that the changes of synaptic efficacies at PF→PC (e.g., [40]) and also PF→IN [38] are important for adaptive cerebellar learning. The IO, as the sole source of CFs, has been regarded as an important module that regulates the behavior of PCs. For example, Mauk and Donegan [62] summarized their hypothesis of the role of CFs as follows: “CF activity is regulated to maintain its equilibrium at which the net strength of Gr→PC synapses remains constant unless an unexpected US is presented or an expected US is omitted.” Their summary emphasizes the small temporal scale dynamics of the cerebellar-IO system, which is important in most motor movements that are of short duration. However, this summary does not include some other aspects of IO influence on the cerebellar network, namely, longer-time scale modulation. One crucial clue can be found from IO lesion studies where the CF signal is no longer available. For example, Cerminara and Rawson [63] found that when CFs were silenced, the PCs increased their firing rate even without a change of the synaptic efficacy at PF→PC pathway. This observation emphasizes the following three facts: (1) an elevated PF activity (e.g., [40]) but not the low frequency PF background activity (0.5±0.2 Hz)[64] may induce the synaptic change at PF→PC; (2) whereas short temporal deviation (addition or omission of firing) of CF activity may contribute to the synaptic changes when PF input is also elevated at the same time [62], (3) it is the prolonged (more than a few seconds to minutes) change in CF firing frequency that controls the PC's tonic firing level by changing an intrinsic spike generator in the PC.

More insights of the roles of the IO can be garnered from the long-term (months to years) effects of an IO lesion (see Figure 10). The long-term studies show that the tonic firing rate of PCs increases quickly after the IO lesion, but the tonic level eventually recovers to its pre-lesion level over a month [46] and stays stable afterwards[47]. Our simulation results indicate that the time constant responsible for the quick increment of the PC firing rate is about 10 s while that of the recovery mechanism is about 4.6 days. These two time scales indicate that there are at least two different CF-controlled mechanisms that regulate the intrinsic firing rate of the PC. Notably, both of them seem to measure the relative CF firing activity compared to its two temporal averages, giving the PC a chance to habituate to the prolonged changes in CF activity. One important conclusion of this very long IO lesion simulation is that the model can generate almost the same time course of the change in PC tonic activity without any changes of synaptic efficacy at PF→PC or PC→IN. This conclusion is consistent with the observation by Cerminara and Rawson [63], who did not find any changes at the PF→PC pathway. Also a recent reversible IO lesion experiment by Horn and his colleagues [65] found that although the inactivation of the IO severely depresses the subjects' motor activities, the motor movement comes back to normal as soon as the chemical wears off, indicating that the absence of CF input does not wipe out previously learned motor memory.

### Comparison to other models

There are at least three prominent models of delay conditioned learning. The first one is the network state dependent model (e.g., Medina et al., 2000). It uses a presumed change of MF input pattern during the CS period and granule cell-Golgi cell (Gr-Gg) network to generate temporally varying PF input patterns to PCs. This model also adopts a Marr[66]-Albus[67] style feed-forward PC model. The idea of the model is reminiscent of the proposed mechanism of the antennal lobes of insects[68] where different clusters of cells fire at different points in time after a presentation of an odor similar to the hypothesized spatiotemporally varying PF firing pattern in a network state dependent model. The processing in the antennal lobe and in the following mushroom body seems to bear some similarity to that of the cerebellum. However it is not clear if the assumed temporally varying cerebellar PF input patterns are necessary for the PCs to generate a timed response. For example, Shinkman et al. [50] emulated the classical conditioning paradigm with electrical stimulation at the surface of cerebellar cortex as a CS (350 ms train of 60 Hz alternating current) and electrical stimulation of the white matter just below the surface electrode as a US (a 100 ms coterminous US). The animals learned the conditioning normally, generating a CR at the time of the PF electrical stimulation. Even with this presumably spatio-temporally uniform PF input, the cerebellar cortex learned the classical conditioning. This indicates that a spatio-temporally varying PF input pattern may not be a prerequisite for the timed expression of the CR. We think that the alternating CS current may have entrained or even dominated the pattern of the PF inputs. Therefore, the mechanism responsible for the cerebellar timing may reside somewhere after PFs. The other two categories of models (spectral timing model, adaptive-PC timing model) are compatible with this assumption.

The spectral timing model by Fiala et al.[18] is interesting because it shows robust performance even with noisy inputs. It assumes an array of different kinetic constants in the metabotropic signal pathway among a PC population. This enables a population of PCs to respond with different latencies at a PF input. This model employs calcium-activated potassium channels as an adaptive component where a PF-CF coupling-induced metabotropic second messenger pathway trains the potassium channels to increase their conductance. The increased potassium channel efficacy was assumed to be responsible for the pause of the PCs, which generated the learned CR response. This sophisticated mechanism, however, it does not explain a set of more recent data. In a series of investigations, Schreurs et al. [21], [22], [23] found that the PCs in cerebellar lobule HVI had significantly smaller potassium conductances in classically conditioned animals compared to those of the untrained animals. Moreover, they found a strong relationship between the level of conditioning and PC dendritic membrane excitability (more learning more excitability); and this relationship was still present 1 month after classical conditioning. Like the spectral timing model, our model has a set of PCs with a wide range of time constants. However, whereas the original spectral timing model learns by depressing the output of the PC, our model learns by increasing the excitability of both PCs and INs to produce the pause.

**Figure 11:**
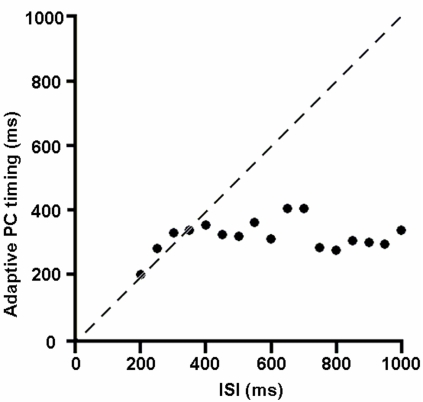
Noise disrupts performance of adaptive-PC timing model. When a more natural activation pattern of noisy CFs was used, its performance dropped at increasing ISIs. This model is vulnerable to intervening noise pulses because it is not based on a large population of neurons. The dashed line indicates the ideal relationship for the data points. See text for details.

The adaptive-PC timing model [19], [20] furthers the concept of the intracellular timing mechanism of the spectral timing model. It proposes that instead of allocating different timing kinetics among PCs, each PC should adapt to the appropriate timing by varying its latency according to the given ISI. It is an interesting concept which may potentially maximize the efficiency of the neuronal resources of the cerebellum. Unfortunately, the adaptive timing model can be confused by the presence of noise in CF firing because it is based on individual, and not population learning of PCs. To demonstrate this point we simulated one of these adaptive-PC models[19]. When noisy CF signals (1 Hz random background CF activation in addition to the US-induced CF signal) were provided, the adaptive-PC timing model showed an increasingly confused response at longer ISIs. This point can be appreciated by the deviation of data points from the dashed line (Figure 11), which designates the ideal alignment between the US timing and the PC's timing. This failure for long ISIs is because the model follows the timing of the PF-CF coupling. The model just decides whether to increase or decrease the latency of PC pause by comparing the current timing of the pause and the timing at which the PF-CF coupling occurs. When the noisy background CF signal is introduced during the ISI period, the model follows the false timing as well as the true timing. This tendency increases as ISI increases, giving more chances for intervening false signals to disrupt the adaptive process. This is an inherent problem in non-population based learning systems because there is just one memory bit available for timing. Population-based models, either granule cell population or PC population, do not have this problem because they have many memory bits spread across time that can register the probability of an event happening at one moment.

### Beyond the model

While the current study focuses on the classical delay eyeblink conditioning, it should be possible to extend our model to explain other phenomena related to cerebellar timing. In *backward* conditioning, for example, the US precedes the CS, and no conditioning response is learned [8]. In this case, our model does not generate a CR either. As is evident from Figure 3, if the CF precedes the PF signal there is no or little regenerative calcium influx in the Purkinje spine. Since the model requires frequent regenerative calcium influx as a prerequisite for the long-time scale learning, the backward coupling will not generate any learned response in the network. In trace conditioning, the CS precedes, and terminates before the onset of the US. It is known that Hippocampus is needed for acquisition, and the medial prefrontal cortex for retention in addition to the cerebellum[69]. Conforming to this, the current model, which only has the cerebellum, does not generate trace conditioning (result not shown). This is because our model requires a significant PF input during the CS period to generate the IP3 envelop for regenerative calcium activation. In case of natural trace conditioning, it is assumed that Hippocampus (during the learning period) or medial prefrontal cortex (during the retention period) provides the needed input for the CS signal in the cerebellum cortex. This situation is beyond the scope of the current model.

**Figure 12:**
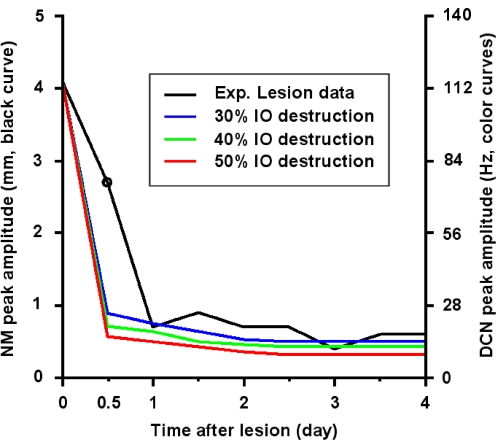
Simulation of IO lesion in classical eyeblink (nictitating membrane) conditioning. The black curve indicates the experimental data from McCormick et al. (1985) after an IO lesion. The first data point at 0 day is the pre-lesion NM peak amplitude (∼4.1 mm) after full acquisition of the classical delay conditioning. Also note that the circled point is the first peak amplitude of nictitating membrane (NM) response in mm (ordinate on the left side) 12 hours after IO lesion. The blue, green and red curves indicate the simulation results of the peak DCN firing rate (in Hz, ordinate on the right side) during test trials with IO destruction percentages of 30%, 40% and 50%, respectively. A noticeable difference is seen for the first trial after IO lesion. The differences between the experimental data and the results of the simulation cannot be explained by the degree of IO lesion. See text for more details.

One puzzling discrepancy in the literature comes from the results of McCormick et al.[70], who did not observe post-IO-lesion motor inactivity in the conditioned response in classically conditioned animals. In that study they examined their hypothesis that the absence of the CF input during the classical delay eyeblink conditioning is equivalent to the omission of the unconditioned stimulus (US). This reasoning led them to examine the effect of an IO lesion in post-acquisition training. They compared the amplitude of the nictitating membrane (NM) responses in the IO-lesioned animals during post-acquisition CS-US coupling (maintenance) trials with that of the normal subjects undergoing CS-only extinction trials. They found statistically similar traces between the two groups in NM amplitudes thus confirming their hypothesis that an IO lesion is equivalent to the omission of the US in classical delay conditioning. The black curve in Figure 12 shows the trace of the NM peak amplitudes during the post-lesion CS-US coupling trials. The conclusion by McCormick et al.[70] showing no post-IO-lesion motor inactivity is in contrast with the well reported depression of the neural activities in DCN (e.g.,[46]) and vestibular nuclei (e.g.,[71]) and the depression of the behavioral counterparts (e.g., [65]).

We examined whether an incomplete IO lesion could explain the results of their study[70] by simulating partial IO destruction. Figure 12 shows the results of the simulation with IO destruction of 30% (blue curve), 40% (green curve) and 50% (red curve). Considering lesions smaller than 30% would be unreasonable, because the lesion in the rostromedial dorsal accessory IO was relatively large in their study (see Fig. 4 in [70]). Furthermore, any increase or decrease of the scale of simulated IO destruction did not change our conclusion (not shown). To compare the simulated DCN activity (Hz) with the behavioral NM amplitude (mm), we linearly rescaled the ordinal axis of the DCN output to match the maximum amplitude of the CR-related DCN output of ∼115 Hz (c.f. Supplementary Figure S2F, see also Note 2 in Text S1) to the maximum NM amplitude (∼4.1 mm). Even after this generous rescaling, which approximately matched the asymptotic tails of the simulation data (especially the blue curve) to the data traces, there was a noticeable discrepancy between the data and the simulation results, especially between the first post-lesion NM amplitude (circle) and those of the DCN activities (the points at 0.5 in blue, green and red curves). This discrepancy indicates that the unabated behavioral strength in the lesioned animal in their study cannot be explained solely by incomplete destruction of IO. It is especially interesting to note that their post-lesion trials were performed about 12 hours after the IO lesion and lasted for a few days. This period of time, according to Batini et al.'s data[46] (see Figure 10), is when the DCN is maximally suppressed due to the increased PC firing rate, thus presumably leading to a minimal motor activity. Maximal suppression suggests that the NM response should be at a minimum, but McCormick et al.[70] found only a slight suppression. Further study is needed to explain this discrepancy in the literature.

It is known that PCs and DCN neurons display burst firing patterns after IO lesions[47]. The current model does not explain this behavior of the cells, which is presumably caused by intrinsic cellular properties[72]. A more biophysically complete model would be needed to address this long-term lesion effect.

It is known that inhibiting the expression of CRs during the extinction training by reversible inactivation of the DCN[73], [74] or even facial nucleus and accessory abducens[75] prevents the extinction of the learned CR. So far there is no concrete explanation why this may be the case. There are several possible explanations one being the permissive action by the anterior lobe[9], [10] mentioned above. Although our model uses a simplistic approach for the reversal of the acquisition, it is likely that many components of the neural circuits contribute to different aspects of extinction[68]. Currently our simplistic mechanism does not explain the findings concerning the reversible inactivations, especially, the inactivation of facial nucleus and accessory abducens. Further investigations are needed to fully account for this issue. For more discussion refer to Kurpa and Thompson[75].

### Suggested experiment

In all other current cerebellar timing models that we know of, INs do not play a significant role in shaping the dynamic firing pattern of PCs. In contrast, the current study proposes that “in long time scale cerebellar timing, it is the interaction between the PC and INs that shapes the waveform of activation in the PC”. According to our hypothesis, the modulation of PC activity over long-time scales should be closely related to that of its paired INs. One example of this related modulation of activities can be seen in a simulated PC-IN pair in Figure 7 middle row. In the figure, the decrement of PC activity is related to the increment of IN activity. Although there have been a few attempts to record PC-IN pairs, especially in short time millisecond scale interactions (e.g. [76]), there is no *in vivo* data available for long-time scale interactions (like the one in Figure 7 middle row). One possible experiment that could examine the validity of our model's prediction is to record PC-IN pairs that show modulation of activity in HVI lobule *in vivo* after acquisition of delay eyeblink conditioning. By examining the form of interaction between PC and IN on a long-time scale, it may be possible to see the source of the timed pause of the PCs, which is deemed to be the mechanism of cerebellar timing.

### Conclusion

Early models called for depression of PC activity to generate a timed response. Recent experiments showed, paradoxically, that excitability of the PCs increased after natural learning. Our model resolves this paradox by suggesting that natural learning causes an mGluR-mediated increment in the excitability of both PCs and INs. Their interaction generates the depressed output of the PC, which makes the pause in the PC's activity at the correct time, as required by cerebellar theory. This simultaneous increase of the mGluR-mediated activity both in the PC and IN for cerebellar timing is consistent with the evidence that (1) mGluR-mediated excitability in the cerebellar cortex contributes to the slow motor movements[26], (2) the kinetics of mGluR in the INs and the PCs of the cerebellar cortex is similar[28] and (3) natural learning of the delay conditioning increases the excitability of the PCs in the Larsell's lobule HVI[21], [22], [23]. This new PC-IN interaction mechanism gives the INs a richer role in cerebellar function than simply as an automatic gain controller for the PCs, as assumed before. In terms of functionality, the suggested mechanism of PC-IN interaction can generate a waveform that can be shaped to match an arbitrary learned output, endowing the cerebellar cortex with the versatility to learn almost any temporal pattern, in addition to those that arise in classical conditioning [30].

## Materials and Methods

First, we will describe the function of each part of the model in plain language. Second, for those interested in the details of the simulation, we will give the formal descriptions and equations of each computational step in our model.

### Mossy fibers

The model simulates the CS by providing an elevated level of MF signal during the CS period as illustrated in Figure 1A (cf. Eq. 1 below). The signal is then relayed to the granule cell (Gr) and Golgi cell (Gg) network and the DCN. This mechanism is based on the experiment by Hesslow et al. [32], where electrical stimulation of MFs mimicked the natural CS. One important result of their experiment is that a spatially and temporally varying MF input pattern is not required for expression of the CR, because there was no such variation in their MF stimulation.

### Granule-Golgi module

The current model adopts the usual gain control hypothesis [37], [66], [67] and uses the Gr-Gg negative feedback circuit (Figure 2A) to normalize the activities in the Gr population. Also, the simulated Gg uses an activity-dependent adjustable excitability [77] to accommodate and remain sensitive to any long-term changes in the input (cf. Eq. 4). This is done by varying the potential value for which the firing rate is half of its maximum value.

### Purkinje cell-inhibitory interneuron module

The simulation has 54 PC-IN pairs that receive parallel fiber inputs. Each PC-IN pair has one PC with two branches and two INs, each inhibiting one PC dendritic branch (PCBr). Each PCBr bears three spines where parallel fibers terminate. Figure 2B illustrates the module showing one pair of IN-PCBr for simplicity. Each inhibitory neuron and its connected PCBr constitute one computational unit in the model. This concept is consistent with the observed lateralized inhibition and plasticity of PCBr, specific to the stimulated side of the beam of parallel fibers [78], [79].

### Computation in Purkinje cell spine and interneuron dendrite

The model adopts the theoretical findings by Doi et al. [39] regarding the signaling mechanisms in the PC spine at PF and CF inputs. They showed that a PF signal generates an IP3 activation profile that acts as a window in which the CF signal can trigger the slow-rising calcium component (Figure 6A). Figure 6B shows the electrochemical circuit in our model that simulates the process of the regenerative calcium release (the thick arrow) from the ER (Eq. 7). The model shows that the PF signal stimulates mGluRs, which in turn activates IP3 (Eq. 11). When this IP3 activation is combined with an elevated level of [Ca^2+^]*_i_* caused by CF→AMPAR activation of the voltage-gated calcium channel (VGCC), it causes positive calcium feedback by releasing calcium ions from the ER (Eq. 17). The big arrowhead with two stems in Figure 6B indicates this multiplicative process. Our model also includes a similar slow-acting calcium activation mechanism in the IN dendrites, because of the recent finding by Karakossian and Otis [28] that both PCs and INs have a slow-acting long-time scale excitatory component mediated by an mGluR pathway.

**Figure 13:**
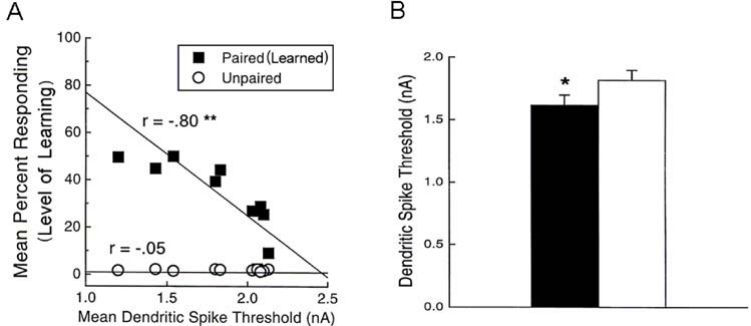
Learning-specific membrane excitability after 1 day of classical conditioning (A), and 1 month after 3 days of classical conditioning (B). A. Strong linear relationship between level of conditioning and mean dendritic spike threshold (at least 2 measures per rabbit) for trained rabbits (*filled squares*, *r* = −0.80, *p*<0.01) relative to control rabbits (*open circles*, *r* = −0.05, *p*>0.8). Note that better trained animals have lower threshold (more excitable) PC dendrites. B. Mean dendritic spike thresholds showing a significantly lower threshold in cells (*n* = 61) from trained rabbits (black bar) than in cells (*n* = 47) from control rabbits (white bar). *p<0.05. Figures are from Schreurs et al. [21].

The ionotropic pathway triggered by the PF signal (PF→AMPAR→VGCC→Ca^2+^ in Figure 6B), before any delay conditioning, is assumed not to be strong enough to trigger long latency calcium activation. It is hypothesized that during the delay conditioning paradigm coupling the PF signal with a delayed CF signal can increase the efficiency of the PF-mediated VGCC pathway. This hypothesis is consistent with the experimental findings by Schreurs et al. [21], who reported a long lasting (at least a month; see Figure 13B) increment of excitability in PC dendrites in lobule HVI that is proportional to the learned response level of the animals as shown in Figure 13A. The mechanism of this increased efficiency is not known. One possibility would be a decrement of the conductivity of potassium channels, as suggested by Schreurs et al. [21]. Another possibility is that the CF-PF coupling may increase the efficacy of the IP3 receptor (IP3R). Potentiation of IP3R has been observed in PC soma [80], but the plasticity of IP3R in the spine of the PC has yet to be established.

One crucial hypothesis of our model is that this slow-acting [Ca^2+^]*_i_* change is involved in both learning and motor execution. This hypothesis is prompted by the results of Coesmans et al. [26], who found that acute deactivation of mGluRs in PCs affected motor execution, especially slow components (low frequencies) of movement. The fact that INs as well as PCs have a long-time scale component suggests that the PC-IN system is symmetric in long-time scale as well as short-time scale features, thus indicating multiple processes can evolve in parallel in the time domain. To implement this hypothesis, a range of time constants governing the latency of IP3 activation are assigned to PC-IN pairs in the model (Eq. 11). This lets the PC-IN pairs have a range of time courses of activation in the mGluR-mediated long-time scale component. To reflect the reported mean peak latency of the slow calcium component [48], [49], the time constants are chosen for IP3s to have a relatively denser representation around 300 ms, but with a broad Gaussian distribution.

**Figure 14:**
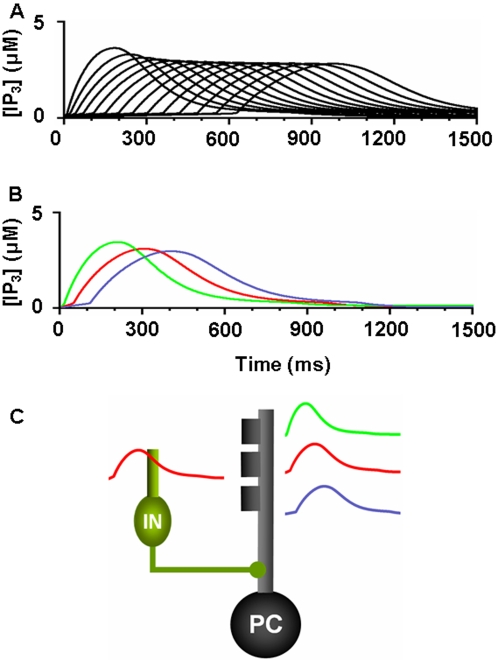
Distribution of IP3 activation profiles for the PC-IN population used in the model. A. IP3 activation in PC-IN population after onset of a PF signal. Each PC-IN pair is assumed to have one main time constant that generates an IP3 profile. The curves show IP3 activations by a PF signal that continued throughout the displayed time period (1500 ms). For clarity, every fourth IP3 component used in the simulation is displayed. It is assumed that the distribution of the peak time has a broad Gaussian distribution with a center around 300 ms, thus having a relatively denser representation at early timing. B. An example of one shared IP3 peak latency (in red) and two small variations (in blue and green) inside of a PC-IN pair. C. A cartoon explanation of the IP3 latencies. In this example, the PC has an IP3 peak latency of 300 ms (red) shared with the IN dendrite. A variation of IP3 peak latencies at 200 ms (green) and 400 ms (blue) are assigned to the other two spines. Note that in this example only the 300 ms peak latency is shared by the PC and IN (red).

Figure 14A shows the simulated IP3 activation profiles upon PF input. For simplicity, only every 4^th^ IP3 activation profile is shown. Whereas only one IP3 peak latency is shared by each PC-IN pair, a variation of ±100 ms in peak latency is also added to simulate variation inside each module. Figure 14B and Figure 14C show an example where a PC-IN pair has one shared IP3 profile at 300 ms (in red) and two flanking profiles (in green and blue, at 200 ms, 400 ms). A cartoon PC-IN pair in Figure 14C shows this configuration where the red IP3 profiles are shared by one of the PC spines and the IN dendrite while the flanking IP3 profiles (in green and blue) are assigned to the other spines. This gives each IN-PCBr unit a relatively broadly-spread activation time course for the PCBr and a focused inhibitory activation for the IN. The reason for the IN to have the temporal envelope centered at the middle of the activation profile of PCBr (see Figure 2B) is to maximize the efficiency of one of the supposed roles of INs, which is to protect the PC from potentially excessive excitation [78], [81]. We found that the exact shapes of these profiles or latencies are not critical (see the section “Timing in Purkinje cell-inhibitory neuron module” for more details). This is also due to the property of the learning rule (see the following section), in that the IN-PCBr system is able to generate a waveform that becomes modulated in proportion to the probability of an event happening in the IP3 time windows of that IN-PCBr module.

### Computation in Purkinje cell dendrite and soma

The dendrite of the PC integrates four inputs: signals from spines, soma, inhibitory interneurons and the CF (Eq. 25). The model dendrite has a passive integration property as suggested by Heck et al. [82]. The parameters for the four input sources are chosen so as to cancel out any elevation of potential during PF input signals when no learning is involved. This is consistent with the finding that feed forward inhibition effectively suppresses a beam of PC activation along the PFs when stimulation is given in the granule cell layer [83]. The simulated soma of the PC integrates the dendritic signals. It also has a tonic component that is under CF signal control (Eq. 27) [63]. This tonic component simulates the role of the CF signal in controlling the baseline firing rate of the PC. It was shown by Cerminara and Rawson [63] that the baseline firing rate was controlled mainly by the ongoing CF signals and that blockage of PF signals had a minor effect on the tonic PC firing rate.

### Learning Rules in Purkinje cell-inhibitory interneuron module

Recent experiments have found that the PF→PC synapses are able to change their strength bidirectionally depending on the presence (resulting in LTD) or absence (resulting in LTP) of a CF signal at the time of a PF signal [40]. It is believed that PF signal-activated nitric oxide (NO) [84] “allows” the learning to happen, and it is the CF-induced voltage-dependent calcium channel activation that decides the direction of synaptic plasticity [40]. Extending this assumed bidirectional (LTP or LTD) synaptic learning mechanism at PF→PC, Rancillac and Crepel [38] observed similar, but opposite sign, learning at PF→IN synapses: LTP after PF-CF coupling, LTD after PF alone. The current model incorporates these observations and implements push-pull type learning between PF→PC (Eq. 8) and PF→IN (Eq. 30) synapses for the short-time scale component. First, the simulated PF→PC (Eq. 8) and PF→IN (Eq. 30) synapses calculate the instantaneous change of postsynaptic [Ca^2+^]*_i_* (denoted as *α*) and its average over a long time (denoted as *β*). The difference (*α*−*β*) averaged over a short time (*∫*(*α−β*)d*t*) indicates whether the CF activity has increased (*α*−*β* over time>0), decreased (*α*−*β* over time<0) or remained the same (*α*−*β* over time = 0) in a given period. By multiplying this difference with the PF signal over time (*PF*×[*α*−*β*] over time) the model then estimates whether the CF activity has increased (>0), decreased (<0) or remained the same ( = 0) at the time of the PF signal. This value determines whether the synaptic strength needs to be updated (cf. Table 1A).

**Table 1:** Learning rules for two different time scales.

A. Short-time scale				
	*PF*×[*α*−*β*] over time:	= 0	>0	<0
Synapse	PF→PC:	no change	LTD	LTP
	PF→IN:	no change	LTP	LTD

B. Long-time scale				
	*IP3*×[*α*−*β*] over time:	= 0	>0	<0
Synapse	PF→PC:	no change	increment of excitability	decrement of excitability
	PF→IN:	no change	increment of excitability	decrement of excitability

For short-time scale, instantaneous change of postsynaptic [Ca^2+^]*_i_* is *α* and its average over a long time is *β*. The difference (*α*−*β*) integrated over a short time (*∫*(*α−β*)d*t*) indicates whether the CF activity has increased (>0), decreased (<0) or remained the same ( = 0). For long-time scale, *α* and *β* are the instantaneous CF signal and its average over a long time, respectively. The terms *increment* or *decrement of excitability* are used for long-time scale plastic changes to discriminate these from the well known LTP, LTD phenomena, which primarily refer to the changes in AMPAR pathways.

For the long-time scale component, the model simply replaces the PF gating signal with the PF signal-induced intracellular concentration of IP3 ([*IP3*]*_i_* in Eq. 21). Because the exact mechanism is unknown, the long-time scale generates the variables *α*, *β* using the instantaneous CF signal and its average over a long time, respectively. This IP3 requirement restricts the time window of synaptic change to the time of IP3 activation, which varies across the PC-IN population. The learning rules are summarized in Table 1B. Note that when the PF-induced IP3 signal is coupled with a temporary increase of the CF signal, an increment of excitability occurs in INs as well as in PCs.

Using the mechanisms summarized in Table 1, the population of PC-IN pairs learns to increase PC output at unpaired PF signals (without CF signals) and decrease PC output at paired PF signals (conjunctive PF-CF activation) via the short-time scale component (LTP/LTD). For the long-time scale component, if the conjunctive PF-CF happens regularly with a specific ISI, the PC-IN population learns to modulate the PCs' firing profiles at the time of the expected CF signal, due to the increased long-time scale activities of the PC-IN pairs. This modulation is limited to the PC-IN pairs that have similar latencies of activation to the ISI (cf. Figure 7). The resulting discharge of the PC population then reflects the modulations in its constituents and shows a timed decrease of firing. The outputs of the PCs are then transmitted to the DCN.

### Deep Cerebellar Nucleus Module

The simulated excitatory deep cerebellar nucleus neuron (DCN) integrates the inhibitory signals from PCs and excitatory signals from MF collaterals and CF collaterals (Figure 2A, Eq. 47). Similar to the learning rules in the cerebellar cortex, the model implements plasticity rules at the MF collaterals→DCN synapses (Eq. 54). If the average of (*MF*×[*γ*−*δ*]) over time>0, then increase the strength (LTP) at MF collateral→DCN synapse. Where *γ* and *δ* represent instantaneous input from the CF collaterals and its time average, respectively, similar to the concept in the learning rules in the cerebellar cortex. If (*MF*×[*γ*−*δ*] over time)<0, then decrease the strength (LTD) at MF collateral→DCN synapse. If (*MF*×[*γ*−*δ*] over time) = 0, do not change strength. As Mauk and his colleagues point out, there is no direct evidence for any known specific rules for MF collateral→DCN synapse (see [85] for recent findings). Some indirect evidence for the CF collateral-induced synaptic change is the configuration of the CF collateral terminals that contact distal as well as proximal dendrites and cell bodies (e.g., [86]). It is also known that CF collaterals may be potent enough to induce spikes in the target DCN neurons (e.g., [87]).

The model deep cerebellar nucleus module also implements the inhibitory projection neuron (IDCN). It is assumed that IDCN has properties similar to DCN. Due to this construction, the IDCN mimics the activation properties of the simulated DCN neuron, letting the DCN module give negative feedback of the cerebellar output to the IO via the IDCN→IO pathway.

### Inferior Olive Module

The simulated inferior olive (IO) module uses spiking compartmental model neurons adapted from Schweighofer et al. ([88], [89]; see Eq. 32, Eq. 42). Each neural unit has a soma and four dendrites (Figure 2A), and the dendrites connect to adjacent dendrites of neighboring neurons via gap junctions (zigzag lines in Figure 2A). The soma compartment has the known intracellular currents such as the low-threshold calcium current and Hodgkin-Huxley type sodium and potassium currents. The dendritic module has a high-threshold inward calcium current, an outward calcium-dependent potassium current and the excitatory and inhibitory input currents from extra-cerebellar projections and the IDCN neuron, respectively.

The IO neurons spike quasi-randomly (cf. Figure S2A, A2B and A2C, in Supporting Information). The randomness comes from the interaction between neurons in the IO network that are communicating through local gap junctions (e.g., [88]). However, the firing pattern of individual neurons is not completely random due to the intrinsic pacemaker mechanism coming from an oscillatory, anomalous inward rectifier current (*I_h_*, e.g., [89]).

### Model Equations

We simulated a population-based model of the cerebellum with multiple layers of cell types. Indices of *i*, *j* are used to indicate the (*x*, *y*) positions of a cell in Cartesian coordinates in one group. More indices, for example *k* and *l*, are used to indicate different groups. In the following, the simulated membrane potentials of cellular components are italicized (e.g., the membrane potential of Gr as *Gr*). The simulated potential is normalized to range from 0 to 1, unless mentioned otherwise. Also, time constants of differential equations are integrated into the parameters in the following equations for simplicity, unless specified otherwise. Before starting the simulation, relatively large time constants were set small and slowly increased to their values listed in the equations below while the whole network was running without CS, US inputs. This annealing process was done to automatically set temporal average variables with large time constants to their baseline values. Supplementary Figure S4 (and Note 4 in Text S1) summarizes the network and the corresponding variables.

### Granule-Golgi Cell Network

The Granule-Golgi cell (Gr-Gg) network has 54 (3×9×2) Grs at (*i*, *j*, *k*) and three Ggs. Each Gg communicates with underlying 18 (3×3×2) Grs. Input signals to Grs are given through 54 mossy-fibers (MFs) as follows:
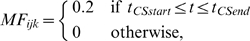
(1)where *t_CSstart_* and *t_CSend_* are the time of the beginning and the end of the CS signal, respectively. Also, a constant background signal of 0.2 was provided via one third of MFs to simulate the vast background input from more than 100,000 synaptic contacts *in vivo*. Each Gr at location (*i*, *j*, *k*) gets its excitatory input from the MF at (*i*, *j*, *k*) and inhibitory input from its overlying Gg. The membrane potential of the Gr is governed by the following equation:
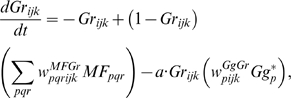
(2)where the first term represents the leakage; the second one, excitatory MF input; the third, inhibitory Gg input. *a* is a constant set to 20 to maintain a low activation rate of Gr at rest (as reported by Chadderton et al. 2004). *Gg* ^*^ represents the firing rate of the Gg with a sigmoidal activation function:
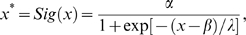
(3)where *α*, *β* and *λ* are constants of 1, 0.2 and 0.08 respectively. 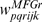 and 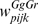 in Eq. (2) are the synaptic weights at *MF*→*Gr* and *Gg*→*Gr*, respectively, and are set to 7 and 0.55. These weights are chosen such that when the MFs transmit the CS signal, the activation level of Grs, which represent the PF signals, reaches high enough for the PC to trigger mGluR activity [49]. The output of Gr, Gr*, is also a sigmoid function, *Sig*(*Gr*), as in Eq. (3) with *α*, *β* and *λ* being 1, 0.6 and 0.12, respectively.

The Golgi cell (Gg) gets inputs from the 18 underlying Grs via the parallel fibers. Gg in turn gives negative feedback to the Grs. The equation for the membrane potential of the Gg is as follows:
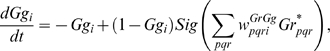
(4)where the first term is leakage, and the second term is the excitatory input from Grs with 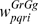 representing the synaptic efficacy at Gr→Gg. The synaptic efficacy is chosen as 0.4 to give Grs a relatively large dynamic range, even under Gg suppression, between rest and CS signal transmission. This relatively large dynamic range made the Gr-Gg network a reasonable gain control system. The function *Sig*(*x*) in Eq. (4) is imposed on the input to accommodate the large number of inputs from Grs. The function has the same sigmoid form as in Eq. (3) with constants *α* and *λ* being 1 and 0.05 respectively. To let the Gg be more adaptive to varying range of input, *β* of the sigmoid function is made a variable as follows:
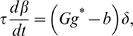
(5)where *τ* is a very long time constant (500 s); *b* is a desired firing rate of Gg during its activation and is set to 0.2. *δ* is a piecewise linear function of the firing rate of Gg as follows:
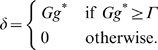
(6)Γ is a threshold set to 0.11. Using Eq. (5) and Eq. (6), the Gg is able to adapt to relatively slow changes in the input to maintain its sensitivity.

### Purkinje Cell

There are 54 (6×9) PC-IN modules in the simulation, each of which has one PC and two INs. The model PC has one soma at (*i*, *j*) and 2 dendritic branches (*i*, *j*, *k*). Each of the branches in turn has 3 spines (*i*, *j*, *k*, *l*) where the parallel fibers terminate. The simulation considers one PC dendritic branch (PCBr) with 3 spines and the corresponding IN as one computational unit (IN-PCBr unit). This means that there are two IN-PCBr units for each PC-IN module. The signal processing in the Purkinje cell spine (PCSp) obeys the following equation:
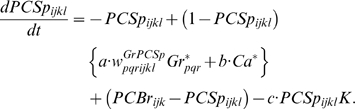
(7)The first term on the right side of equation is a leakage term; the second term represents the excitatory process with the first term in the bracket expressing the input from the Gr and the second, the contribution of calcium-induced potentials, such as via a Na^+^/Ca^2+^ exchanger and a slow excitatory postsynaptic current [90]; the third term describes the potential due to the current between the spine and the connected dendritic branch of the PC (PCBr); the last term expresses the contribution of the potassium channel. The constant *a* is set to 10 to ensure that the potential of PCBr, described in Eq. (25) below, stays the same on CS-induced PF input, balancing the feed-forward inhibition from the attached IN during the initial stage of PF-CF coupling [83]. *b* of 2.5 is chosen to simulate the assumed increase of potential due to the concentration change of Ca^2+^ [18]. The constant *c* of 0.5 is chosen to block any prolonged positive feedback between the voltage-activated calcium channel described in Eq. (17) and the potential of the PCSp.

Two adaptive processes are located in the spine: the fast synaptic efficacy at AMPA receptors (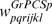), and the metabotropic receptor efficacy and ensuing IP3 activation mediated [Ca^2+^]*_i_* change. The synaptic weight between Gr and PCSp changes with the following rule:
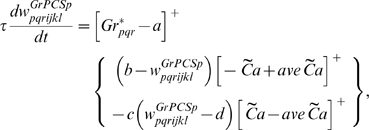
(8)where *τ* is a time constant of 33 ms, and *b* and *d* are the upper and lower bounds of the synaptic weight (0.9 and 0.2, respectively). The constant *c* of 2.5 is introduced to set a reasonable baseline weight. The bracket with a plus sign, [ ]^+^, indicates a positive-value-only rectification process. A small constant of 0.004 is chosen for *a* to ignore small amounts of noise. *C* ~*a* is the calcium fluctuation defined as follows:
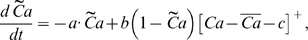
(9)where *a*, *b* and *c* are constants of 0.3, 200 and 0.03, respectively. 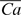 and *aveC* ~*a* are temporal averages of the calcium concentration and its fluctuations, respectively, and result from the following averaging process:
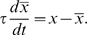
(10)
*τ* values for 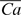 and *aveC* ~*a* are 1000 s and 2 ms, respectively. Using Eq. (8) the synapse detects any fast positive or negative fluctuation of calcium concentration. Since Eq. (8) is a multiplication between the activity of the granule cell output (the first bracket) and negative (first term in the large bracket) or positive (second term in the large bracket) calcium fluctuations, this implements the concept of parallel fiber signal coupling with the postsynaptic change of calcium concentration. Here the change of calcium in time is emphasized to prevent a prolonged change of calcium concentration from dominating the change of synaptic efficacy.

A simplified mechanism is used to simulate the change of calcium concentration upon PF excitation. It starts with a concentration change of IP3 as follows:
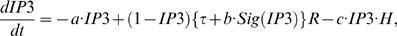
(11)where *a*, *b* and *c* are constants of 10^−2^, 5×10^−2^ and 3 respectively. The subscript *ijkl* of *IP3* that corresponds to the one in PCSp*_ijkl_* has been omitted for clarity. In the following, the same simplification will be made for other intracellular processes. *Sig*(*IP*3), which gives a positive feedback mechanism, is a sigmoid function as in Eq. (3) with *β* and *λ* being 0.05 and 3×10^−3^, respectively. The asymptote of the sigmoid function, *α*, is set to 1.4*τ* ^0.1^ to simulate the smooth decrement of *IP*3 peak values as a function of *τ* as shown in Figure 14A [18]. To simulate the timing property of the cerebellar network, a range of *τ* values were distributed among spines of the PC population. This was done by choosing *τ* values for the peaks of IP3 from a Gaussian distribution centered at 300 ms in PC population (Figure 14A).

The IP3 peak latencies among the PC population are determined as follows:
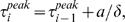
(12)where *a* is a constant of 34.6 ms. The *i* refers to the index of the PC-IN pair and ranges from 1 to 54. The above equation defines the relation between adjacent 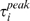 in a recursive form with the first component 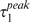 being 180 ms [49]. *δ* in the equation is a density function defined as follows:
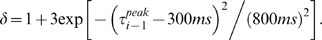
(13)For simulation purpose, the time constants *τ* were calculated before simulation by feeding the CS signal to the system and measuring the IP3 peak latencies and then adjusting *τ* until every IP3 peak occurred at the designated 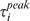. For simplicity, peak latencies over 1 sec are not included in the simulation. Each IN-PCBr unit has one main IP3 peak latency (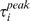) that is shared by one PCSp and the IN. The other two PCSps of the unit have 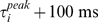 and 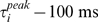. For example, one IN-PCBr unit may have three PC spines with IP3 peak latencies 200 ms, 300 ms, 400 ms and the attached IN has an IP3 peak latency of 300 ms. The other IN-PCBr unit of the PC-IN triplet has the same composition of IP3 peak latencies for its constituents. *R* in Eq. (11) acts as an excitatory signal for IP3 and represents the activation of mGluR1 upon PF input:
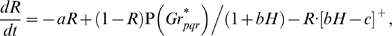
(14)where the decay constant *a* is set to 0.03 to simulate the long lasting kinetics of the mGluR-mediated signal cascade. *b* and *c* are constants of 150 and 1.5 respectively. The function 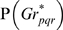 is a piecewise function that implements the fact that mGluR activation requires a significant excitation by PF input:
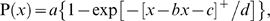
(15)where *a* and *d* are constants of 4 and 0.002, respectively. The constants *b* of 1.3 and *c* of 0.04 are chosen to shift the dynamic range of the P(*x*) function such that a prolonged exposure to elevated input desensitizes the mGluR, implementing habituation. *x̅* is an average that obeys Eq. (10) with a very long time constant (10^5^ seconds). *H* in Eq. (11) and Eq. (14) represents a process whereby *IP3* kinase terminates the *IP3* signal by converting it to *IP4* (Schell et al. 2001):
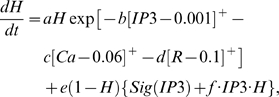
(16)where the first term implements a decay component with the speed given by *a* of 2. This term is multiplied by an exponential function that represents a negative feedback property similar to a reported functional coupling between antagonistic channels [91]. In their recent study, Jow et al. [91] found that increased [Ca^2+^]*_i_* slowed down the inactivation of potassium channels to regulate neuronal excitability. It is hypothesized that there are three components that can influence the decay function with weighting parameters *b*, *c* and *d* of 400, 50 and 50, respectively. The last term with an efficacy coefficient *e* of 8×10^−4^ is a facilitatory term of *H* with a gain *f* of 100. *Sig*(*IP*3) is a sigmoid function as in Eq. (3) with *α*, *β* and *λ* being 1, 0.6 and 0.05, respectively. Because the exact mechanisms of interaction between *IP3* and *IP3* kinase are unknown, we adjusted the excitatory function of *H* so that it reproduced the known dynamics of IP3. With the sigmoidal function of *IP3* and the gating of *IP3* in the activation of *H*, the excitatory term acts as a gated negative feedback circuit. Using Eq. (11) to Eq. (16), the profile of IP3 activation was simulated as in Figure 3D. We simulated the calcium activation at PF-CF coupling with a simplified model of intracellular calcium dynamics:
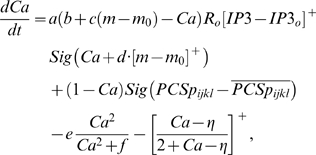
(17)where *a* of 40 is chosen to reliably initiate the slow calcium activity when the gate represented by *R_o_* is open (see below). *b* of 0.5 and *c* of 1.5 set the upper bound of slow-acting calcium activation. *d* of 0.2 and the multiplied term [*m−m*_0_]^+^ implement the model's hypothesis that the slow acting calcium component is adaptive with an increment of efficacy at CS-US coupling at the correct timing represented by 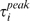 explained above. As the value *m* increases, a smaller increase of calcium (*Ca* in the equation) will be enough to trigger the slow component of calcium activation. IP3*_0_* of 0.05 is given as a threshold value for IP3. The constant *m_0_* set to 0.3 is a baseline efficacy of slow calcium activation represented by *m* (see below). The first *Sig*(*x*) is a sigmoid function with *α*, *β* and *λ* being 1, 5×10^−2^ and 5×10^−3^, respectively. The second sigmoid function of the equation, which describes the voltage-gated calcium dynamics, has parameters of 0.7, 0.3 and 0.01 for *α*, *β* and *λ*, respectively. These values for the voltage-gated calcium component are chosen for the PCSp to trigger the slow-acting calcium component only at IP3-CF coactivation [39] during initial stage of learning. 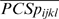 is an average of the potential of the spine with a time constant of 100 seconds. The last two terms with constants *e* and *f* of 0.2 and 0.2 simulate the ATPase pump at the ER membrane and Na^+^/Ca^2+^ exchanger at the plasma membrane, respectively [18]. *η* in the equation is defined as follows:
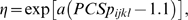
(18)where the constant *a* of 3.9 is 100*F/RT* with *F*, *R* and *T* being the Faraday constant, the gas constant, and thermodynamic temperature, respectively.

*R_o_* in Eq. (17) describes the gating of the slowing-acting calcium channel:
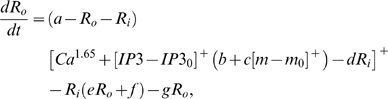
(19)where *a* is the upper-bound of 0.1. The calcium component in the bracket explains the positive feedback mechanism [18]. The multiplied term between IP3 and [*m−m*_0_]^+^ is to implement the hypothesis that the CS-US coupling may increase the efficacy of the slow component calcium kinetics (see below) with the baseline efficacy factor *b* and the coupling-induced contribution coefficient *c* of 1×10^−3^ and 0.1, respectively. The rest components represent negative feedback mechanisms similar to those in Fiala et al. [18] with *d*, *e*, *f* and *g* of 10, 10, 0.11 and 0.486, respectively. *R_i_* represents the closed state of *IP3* receptors [18] as follows:
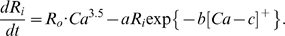
(20)The constants *a*, *b* and *c* are 0.1, 70 and 0.06, respectively. Using Eq. (19) and Eq. (20), the gating system generates the slow regenerative calcium profile in the PC spine at relatively high excitatory input from PF. It is assumed that the efficacy of the slow calcium activation, represented by *m* in Eq. (17), is adaptive where frequent PF-CF couplings result in an increased efficacy as follows:
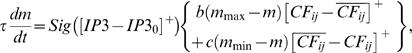
(21)where *m_max_* and *m_min_* are upper and lower bounds of *m* of 4 and −1.286 respectively. The weighting factors *b* and *c* are 0.3 and 0.7, respectively. The parameters *m*_max_, *m*_min_, *b* and *c*, which determine the steady state value *m*, are chosen such that the initial pre-training steady state value of *m* becomes the baseline efficacy *m_0_* explained above. *τ* is a time constant of 200 msec. The function *Sig*(*x*) above, which makes the temporal shape of the IP3 gating signal more Gaussian-like, is a sigmoid function with 1, 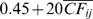 and 0.05 for *α*, *β* and *λ*, respectively. *CF_ij_* and 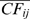 represent CF signal and its average coming from IO at (*i*, *j*). The temporal average of the CF signal is calculated as follows:
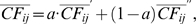
(22)where *a* is a weighting factor of 0.5. 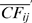 and 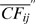 are short and long temporal averages [46], [63]. With Eq. (21), the system increases the efficacy of the long-time scale calcium positive feedback, *m*, if the PF-CF coupling happens (represented by the first term in the large bracket) during the period of IP3 activation (the sigmoid term). If the coupling does not happen during the IP3 period, *m* will decrease (the second term in the large bracket).

The PCSp also has potassium channels. The calcium-activated potassium dynamics is simulated as follows:
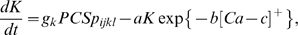
(23)where *a*, *b* and *c* are constants of 1.2, 400 and 0.03, respectively. The conductance of the potassium channels depends on the [Ca^2+^]*_i_* (adapted from Fiala et al. [18]):
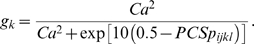
(24)


Each of the simulated Purkinje dendritic branches (PCBr) gets excitatory inputs from the attached spines via conduction currents and an inhibitory input from one IN. The membrane potential of the dendrite is simulated as follows:
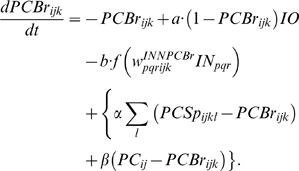
(25)The first term on the right side of the equation is a leakage term; the second term represents the climbing fiber influence with a conduction delay of 3 ms [92]; the third term describes the inhibitory influence by the GABAergic interneurons with *f*(*x*) being *x*(1+*Sig*(x)) to simulate the strong influence of inhibitory input. The sigmoid has parameters of 1.5, 0.075 and 0.01 for *α*, *β* and *λ*, respectively. The two terms in the large bracket represent the conduction processes between the dendrite and the connected spines and the soma, respectively. The constants *a*, *b*, *α* and *β* are 8.5, 10, 1.875 and 1.5, respectively. 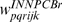 is the synaptic weight between IN and PCBr, and has a fixed strength of 0.25 for simplicity.

The soma of the model PC gets its inputs from its two dendrites. Its membrane potential is described as follows:
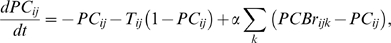
(26)where the first term is the leakage component; the second term represents the tonic component of the cell; the last part describes the potential due to the current between the soma and the attached dendrites. *α* is the conductivity between the dendrite and the soma, set to 1.5. *T* is a tonic component that influences the PC's baseline firing rate as follows:
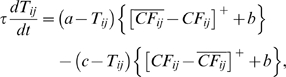
(27)where *a* and *c* are maximum and minimum possible values of *T_ij_* set to 4 and −2.28, respectively; *b* is a small constant, 0.001; *τ* is a time constant of 20 s. The constants are chosen to simulate the short-term and long-term effects of IO lesion on PC firing rate [46].

A linear rescaling factor of 230 Hz multiplied the PC potential described in Eq. (26) to remap the normalized (ranging from 0 to 1) potential, *PC_ij_*, to the spike rates, PC_ij_*:
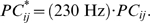
(28)This remapping allowed the baseline potential of a PC to match the reported tonic firing rate of ∼46 Hz [46].

### Cortical Interneuron

One model interneuron (IN) inhibits one of the two PC dendrites, therefore two INs are assigned to each PC. The model IN gets inputs from the Grs via parallel fibers. Inferior Olive (IO) also provides an input to the IN via climbing fibers. The model IN is composed of one dendrite and one soma. The membrane potential of the IN dendritic branch (INBr) is simulated as follows:
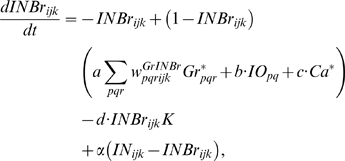
(29)where the first term on the right side of the equation describes the leakage component; the excitatory components are represented in the second term with the inputs from Gr and IO and the influence of calcium-activated processes. The third term represents the inhibitory effect of the potassium current. The last term describes the potential due to the conduction between the branch and the soma. The parameters *a*, *b*, *c*, *d*, and *α* support the same sets of terms in Eq. (7), and have the values of 3.2, 0.53, 3, 0.5 and 1.5, respectively. The weight of the synapse, which represents the efficacy of the AMPAR channel at Gr→INBr, changes with the following learning rule:
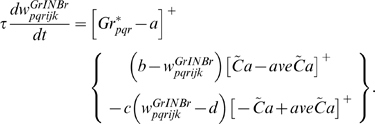
(30)Note that Eq. (30) has the same structure as Eq. (8) with the opposite direction of change. The parameters *a*, *b*, *c*, *d* and *τ* also support the same roles as the ones in the Gr→PCSp synapse with a similar set of values of 0.004, 0.8, 2, 0.2 and 33 ms, respectively. *C* ~*a* is the calcium fluctuation and also follows Eq. (9).

To account for the slowly activating calcium mechanism in the interneurons, which is similar to that of the PC[28], we use the same set of equations (Eq. 11–Eq. 24) used for the PC spine. This simulates the mGluR1 mediated IP3 generation and the ensuing change of calcium concentration at PF input. One adjustment was made in the voltage-gated calcium component: the second sigmoid of Eq. (17) is replaced with 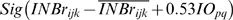 with the sigmoid parameters of 0.65, 0.43 and 0.01 for *α*, *β* and *λ*, respectively. As in the PCSp, the slow regenerative calcium mechanism is assumed to be adaptive and follows the same equation as in Eq. (21). The potassium influence, the third term on the right side of Eq. (29), is also assumed to follow the same mechanism described in Eq. (23) and Eq. (24). Using Eq. (7) and Eq. (29), the PC-IN module creates symmetry in the slow and fast signal processing streams.

The soma of IN integrates signals from its dendrite as follows:
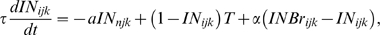
(31)where *τ* (1 ms) [93] is a time constant. The first term is a leakage term with a leakage rate *a* of 0.5; second term represents a tonic component [94] with a constant *T* being 0.035, and the last term is a potential due to the conduction between the soma and dendrite with a conductance *α* of 1.5.

### Inferior Olive Network

The model inferior olive (IO) is composed of a population of 54 (6×9) neural units in a 2D grid with electrotonic coupling between them. Each simulated IO neuron is a compartmental model having five modules representing four dendrites at (*i*, *j*, *k*) and a soma (*i*, *j*). Using the four dendrites each IO neuron contacts four adjacent neighbors [(*i*−1, *j*), (*i*+1, *j*), (*i*, *j*−1), (*i*, *j*+1)]. On the edges of the grid where there are no neighbors to connect, the IO cells still have dendrites with no connections with neighbors. This is to give the soma the same microscopic environment in the simulation. Most of the formulas for the IO network are adopted from Schweighofer et al. [88], [89]. In the following, mostly the modified or typographical error corrected (Eq. (37), Eq. (44), Eq. (45)) parts and their related equations are described in detail. For more details and discussion, see Schweighofer et al. [88], [89]. The potential (in *mV*) of the dendrite module is described as follows:
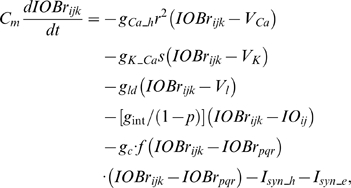
(32)where the terms on the right side of the equation are the calcium current, potassium current, leakage across the membrane, current flowing into the somatic compartment, current flowing in from electrical coupling, inhibitory synaptic current and excitatory synaptic current, respectively (currents in µA/cm^2^). *C_m_* is the membrane capacitance (1 µF/cm^2^). *f*(*x*) represents the transjunctional voltage dependence of the gap junction conductance; *IO* is the membrane potential of the soma; *IOBr_pq_* is the dendritic membrane potential of another IO cell in electrotonic contact with the cell. *g_Ca_h_* (4.0), *g_K_Ca_* (35), *g_ld_* (0.015) and *g_c_* (0.1) are maximal conductances (in mS/cm^2^). *g_int_* (0.13) and *p* (0.14) are constants reflecting the cell morphology for the conduction between the dendrite and soma. *V_Ca_* (120), *V_K_* (−75), *V_l_* (−63; Manor et al. 1997) are the reversal potentials of the calcium, potassium, and leakage currents, respectively. *r* and *s* are activation and inactivation variables, with *r* being defined as follows:
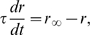
(33)where *τ* and *r_∞_* are the time constant and steady state value of *r*, respectively. They are functions of membrane potential as follows:
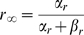
(34)and
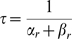
(35)with
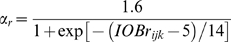
(36)

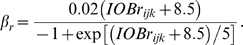
(37)Eq. (37) is adopted from [95]. The excitatory (*I_syn_e_*) and inhibitory (*I_syn_h_*) synaptic inputs from the glomerulus are described as follows:
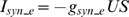
(38)and
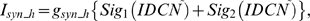
(39)where *Sig_1_*(*x*) and *Sig_2_*(*x*) are sigmoid functions as in Eq. (3) with *α*, *β* and *λ* being 0.15, 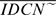, 0.02 and 0.35, 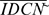, 0.3 respectively. *IDCN* ^∼^ is the trace of inhibitory input from the deep cerebellar inhibitory projection neuron (IDCN) to the glomerulus. And, it is a short-time scale temporal average (see Eq. (10)) with a time constant of 100 ms. The input from IDCN to IO is delayed by 30 ms to reflect the reported delayed action of the inhibition on IO [36]. 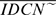 is an average of the trace of inhibitory input (*IDCN* ^∼^) with a very long time constant (10^5^ s). The excitatory synaptic conductance *g_syn_e_* is fixed to 0.31. *US* is the excitatory input with the following rule:
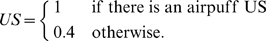
(40)The constant 0.4 at rest is to simulate the baseline glutamate inputs to IO. The inhibitory synaptic conductance *g_syn_h_* is assumed to be adaptive to account for the hypothesized role of the inhibitory DCN input in regulating the firing rate of IO neurons (e.g., [96]):
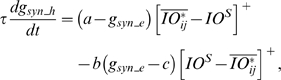
(41)where *τ*, *a*, *b* and *c* are constants of 200 s, 0.8, 1.3 and 10^−3^, respectively. 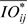 is an average (as defined in Eq. (10)) of the firing of an IO neuron with a time constant of 100 msec. *IO^s^* is the expected average value of 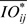 and is set to 0.0031. Using this feedback mechanism, the system tries to keep the firing rate of the IO neuron at an optimal level.

The soma of the model IO neuron is connected to the four dendrite modules. The potential of the soma is described as follows:
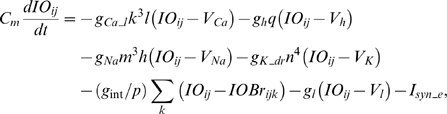
(42)where the terms on the right side of the equation include the low-threshold calcium current, anomalous inward rectifier current, an inward sodium current, a delayed rectifier outward potassium current, the current flowing out into the dendrites, leakage current and the excitatory input current by US, respectively. *C_m_* is the membrane capacitance (1 µF/cm^2^). *g_h_* (1.5), *g_Na_* (70), *g_K_dr_* (18), and *g_l_* (0.015) are maximal conductances (in mS/cm^2^). *k*, *l*, *q*, *m*, *h*, and *n* are activation and inactivation variables. The maximal low-threshold calcium conductance *g_Ca_l_*, is randomly varied between 2.32–2.8 in the IO population to simulate different frequency subthreshold oscillations and firing patterns among individual cells. The kinetics equation of *l* is as follows:
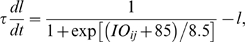
(43)where *τ* is a function of membrane potential:
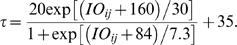
(44)See Eq. (3) and Figure 1B in Manor et al. [97] for details. The kinetics equation of *q* is as follows [98]:
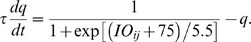
(45)Refer to Schweighofer et al. [89] for descriptions of other variables. *V_h_* (−43) and *V_Na_* (55) are the reversal potentials of the *h* current and sodium current, respectively (potentials in mV). The excitatory synaptic current *I_syn_e_* follows the same rule as in Eq. (38) with the conductance *g_syn_e_* = 0.31.

To match the format of the output of IO (in *mV*) to that of the rest of the network, which uses a normalized value (from 0 to 1) for membrane potential, the output of the IO neuron is calculated with the following normalization process:
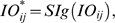
(46)where *Sig*(*x*) represents the sigmoid function as in Eq. (3) with *α*, *β* and *λ* being 1, −10 and 0.01, respectively.

### Deep cerebellar nucleus network

The model deep cerebellar nucleus is composed of one excitatory neuron (DCN) and one inhibitory neuron (IDCN). Both of them receive two excitatory sources of inputs from all MFs and IOs and inhibitory inputs from all PCs. The membrane potential of the DCN neuron is simulated as follows:
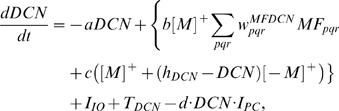
(47)where the first term on the right side of the equation represents leakage speed with *a* = 0.3. The terms in the large bracket explain the gating of the MF input by the PCs activity (first term in the bracket) and the modulation of the intrinsic firing rate of DCN by the PCs activity (the other terms)[59]. More specifically, [*M*]+ implements the rebound mechanism of the DCN, and (*h_DCN_*−*DCN*)[−*M*]+ implements the initial suppression due to the PC inhibition with *h_DCN_* indicating a lower-bound of DCN potential set to −0.25. The gating factor *b* and the modulation constant *c* are set to 0.3 and 0.24, respectively. The third term is the input from IO defined as follows:
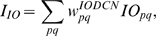
(48)where 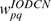 is a connection weight set to 0.03. *T_DCN_* in Eq. (47) is a tonic component that contributes to the baseline firing rate and is set to 0.615. The last term describes the inhibitory influence by PCs with a large temporal scale that measures an absolute level of input from the PC population:
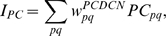
(49)where 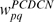 is the synaptic efficacy at PC→DCN (see below). The contribution of this component is given by *d* of 0.6. The hypothesized PC modulation of MF input and DCN potential described in the large bracket in Eq. (47) is consistent with the observations that the amplitudes of learned CRs decline after cerebellar cortex lesion [99], [100], [101]. The PC modulation of DCN represented by *M* measures the deviation of the PC population output from its mean:
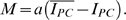
(50)The modulation constant *a* of 4 is chosen to explain the learned CR-related peak DCN firing amplitude of around 110 Hz [44] (see below for potential-to-firing rate conversion). 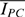 is a weighted average input from PC population defined as follows:
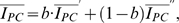
(51)The two temporal averages of inputs from PC population, 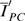 and 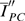, have a short time constant of 100 msec [102] and an intermediate-time constant 7×10^6^ s, respectively, with a weighting factor *b* of 0.4. Theses small and intermediate-scale temporal averages are the bases of the rebound depolarization [103] and the long term decrement of PC control over DCN [47], respectively, via the modulatory mechanism *M* described above. Thus, *b = 0.4* is a compromise between *b = 0*, no rebound depolarization, and *b = 1*, only rebound depolarization.


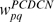
 in Eq. (49) is the synaptic efficacy at PC→DCN and obeys the following learning rule:
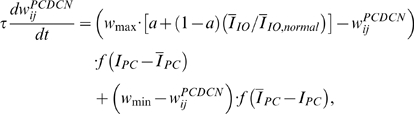
(52)where *w_max_* and *w_min_* are the upper and lower bounds of the synaptic weight set to 0.14 and 0.06, respectively; the weighting factor *a* is chosen as 0.7. These two parameters were chosen to reflect the slow decrement of PC→DCN synaptic weight reported by Billard and Daniel[47]. *I̅* *_IO_* and *I̅* *_IO,normal_* are the temporal averages of the inputs from IO and the temporal averages of the inputs from prelesion state IO, respectively, with a time constant 10^7^ s for both. This multiplication of IO average input and its long time constant are to explain the observed slow decrease of PC influence on the DCN after an IO lesion [46], [47]. When the IO is in its normal state, the multiplication factor will be one (*I̅* *_IO_*/*I̅*_IO,normal_ = 1) and therefore it will have no influence. When the IO sustains a lesion, however, the multiplication factor will decrease slowly, thus explaining the observed decrement of PC control over DCN neurons. The time constant *τ* was set to 3.2×10^5^ s to explain the time course of change of PC influence on DCN neurons [46], [47]. *f*(*x*) is a squashing function as follows:
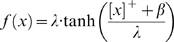
(53)with an asymptote *λ* and a bias *β* being 0.13 and 0.04, respectively. With Eq. (52) the PC→DCN synapse increases (first multiplied terms) or decreases (second multiplied terms) its efficacy when the activity of PC population increases or decreases, respectively, for a prolonged period of time.


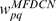
 in Eq. (47) is the synaptic efficacy at MF→DCN with the following learning rule:
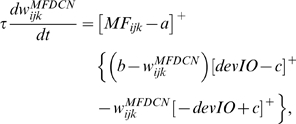
(54)where *τ*, *a*, *b* and *c* are constants of 2 s, 0.05, 0.5 and 0.1, respectively. *devIO* describes the fluctuation in the inputs from IO as follows:
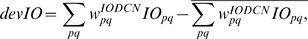
(55)where the second term is a temporal average of the inputs from IO with a very long time constant (10^3^ s). To explain the small transmission efficacy between MF and DCN neurons for non-learned stimuli [14], the initial synaptic efficacy of 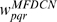 was set to a small value of 0.01.

The mapping of the DCN potential described in Eq. (47) to a firing frequency (indicated by DCN* in the following) is performed by multiplying by 165 Hz:
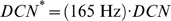
(56)This linear mapping gave a good approximation to the known CR-related DCN firing amplitude in classical eyeblink conditioning, and it also explains the reported baseline firing rate of DCN neurons (∼33 Hz [46]).

Deep cerebellar nuclei also have projection neurons that provide inhibitory inputs to IO. The simplified form of the inhibitory DCN neuron (IDCN) has an equation to that of the excitatory neuron:
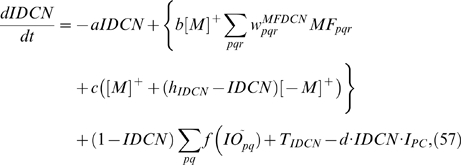
(57)where *a*, *b* and *c* have the same values of 0.3, 0.3 and 0.24 as in the equation for the DCN neuron. The parameters *T_IDCN_* and *d* represent the tonic component and the contribution of the large modulatory PC input component, as in the equation for the DCN neuron, with the same values of 0.615 and 0.6, respectively. 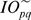 above describes a short-time scale temporal average of 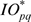:
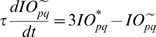
(58)with the time constant *τ* being 10 ms. *f*(*x*) in Eq. (57) is a function of 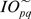 with Hill's coefficient 2:
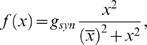
(59)where *x̅* is a temporal average with a very long time constant (10^3^ ms). The maximum conductivity *g_syn_* is set to 0.0015. Using Eq. (58) and Eq. (59) the system maintains its keen sensitivity to the inputs from the IO. Eq. (57) implements the hypothesis that the IDCN may have activity similar to that of the DCN, thus giving negative feedback of the output of the cerebellum to the IO system.

## Supporting Information

Text S1 (0.14 MB DOC)

Figure S1 Conditioned responses of the DCN with (A) or without (B) short-time scale component (LTP/LTD). Panels A and B show that LTP/LTD do not contribute to the conditioned eye blink response. Some differences in the traces between the two figures are due to the initial small random variations in the parameters of the IO neurons used to simulate the natural firing frequency differences between neurons. This small randomness in turn generates some simulation-to-simulation differences in Purkinje and DCN activities.
(0.28 MB TIF)

Figure S2 Normal behavior of model. A. Firing activity of IO neurons and the effect of excitatory input. The point when an external input is given is indicated by an arrow. The neurons quasi-randomly fire with patch synchronization (indicated by circles) when there is no external excitatory input. When an external input is given, the neurons fire synchronously (see the peak in spike histogram at the bottom). Note that the stimulus-induced synchronized firing does not happen for every IO neuron. B. Example of stimulus-induced firing. This example shows spiking by an IO neuron when an excitatory input is given. Note the delay of tens of msec. C. Example of failed firing at external input. This example shows that IO firing is highly dependent on the internal state of individual neurons. The external stimulus just resets the subthreshold oscillation (see the arrow) without driving it enough to spike. D. PC weighted input to DCN neuron. Note that even with the synchronous discharges of IO neurons the output of PC population does not change significantly (arrow). E. Normal state DCN activity. When there is no input to the IO, the firing activity of IO neurons is asynchronous, giving background noisy input to DCN. Even when there is some level of IO synchrony in firing, its impact on DCN discharge is limited (arrow). This fact becomes clear when the firing rate of a DCN neuron during a classically conditioned response is considered (F). F. Learned DCN activity during a CR phase in classical delay conditioning. Comparing the CR-related amplitude with the amplitude caused by the moderately synchronized IO activity makes it clear that ordinary movements require relatively large output from the DCN. Note that the large CR-related amplitude of the DCN activity has been learned by the cerebellar network with the same kind of synchronized IO activity as the one shown in (A).
(0.32 MB TIF)

Figure S3 The correspondence of the lumped model to the original model (A) and internal variables (B). A. The lumped model's behavior (PC: red, DCN: blue) matches to that of the original model (PC: thick orange, DCN: thick cyan) over one day of simulation. B. DCN neuron's internal variables that shape the DCN firing behavior. Black curve: rescaled (×10) synaptic efficacy at PC⇒DCN. Brown curve: weighted input from the PC population to DCN neuron. Purple curve: temporal average of weighted PC population input. Green curve: the medium-scale modulatory component (brown curve - purple curve).
(0.11 MB TIF)

Figure S4 Variables in corresponding anatomical modules in the model are shown. The numbers in the parentheses correspond to the equation numbers in the text. M in the DCN indicates the modulation by the PCs.
(0.95 MB TIF)
