# Supplementary material for: Interaction between Purkinje Cells and Inhibitory Interneurons May Create Adjustable Output Waveforms to Generate Timed Cerebellar Output
Source: PLoS One. 2008 Jul 23;3(7):e2770. doi: 10.1371/journal.pone.0002770 (PMC2474676; doi:10.1371/journal.pone.0002770)
Supplement: Text S1 — (0.14 MB DOC) [file pone.0002770.s001.doc]

**SUPPORTING INFORMATION**

***Note 1. Contribution of short-time scale component***

Unlike our initial expectation, which envisioned some weighted contributions from both scales, we did not see any significant contribution of the short time scale in learned cerebellar timing (Supplementary Figure S1). See text for further explanations.

***Note 2. Normal behavior of the cerebellar network***

Supplementary Figure S2 shows three groups of neurons in the simulated cerebellar network in a healthy state. The IO population gets an external input at time 2 sec as indicated by an arrow in Supplementary Figure S2A. The signal triggers spikes in some IO cells that are in an upswing phase of their potential. An example of this upswing is shown in Figure S2B, where an external input (arrow in the figure) triggers a spike. Typically, when an IO neuron is in a downswing phase of its potential, the external input does not trigger a spike. One example of this failure is shown in Figure S2C (arrow). This behavior of the simulated IO neuron is consistent with the observation by Lampl and Yarom [1] that subthreshold oscillations of the potential of IO neurons can alter the apparent synaptic delay over a range of tens of milliseconds. This property of IO neurons, which originates from their intrinsic subthreshold oscillation, makes their response to an external input stochastic.

Figure S2D shows PC population output as a postsynaptic weighted sum. As expected, the ongoing CF signals do not significantly affect the discharge of the PC population because of the desynchronized nature of the firing pattern among CF (see the raster of the IO firing in Figure S2A). Small patches of synchronized spikes in the raster (circles in Figure S2A) are consistent with the observation by Lang et al. [2] *in vivo*. When an external input is given to the IO, however, it changes the firing probability of the IO neurons and synchronizes the firing of many IO neurons (see the raster and the histogram at 2 sec Figure S2A). This results in a small but noticeable peak in PC population activity (arrow in Figure S2D), which in turn makes a dip in DCN activity (arrow in Figure S2E). This increase in PC population discharge is assumed to be too small to make any significant deviation in the firing rate of DCN neuron to generate any motor movement. For comparison, a simulation result of classical delay eyeblink conditioning is provided in Figure S2F (the same figure as in Figure 8 CR with ISI 250 ms), showing the much bigger amplitude of DCN activity needed to cause a blink.

***Note 3. Lumped model of the cerebellum for long-term (>1000 days) lesion study***

To simulate the long-term (>1000 days) effect of an IO lesion on the cerebellar network, we built a lumped version of the model with only one PC and one DCN cell. This simplification was possible because the lesion study silenced the CF input of the system, leaving only the constant level of background input from MFs. This disconnection of the CFs removed any variability in PCs, such as the complex spikes, making it possible to lump the activity of the PC population together. We also verified the equivalence of the two models by following the trajectory of the PC population output and DCN activity of the original model up to one day in simulation time after lesion (see Supplementary Figure S3).

The IO lesion affects the baseline activity of the PC in the model by changing the tonic component (or “intrinsic spike generator”) of the PC. The influence of the CF input on the tonic component can be summarized with the following rules: (spike generator rule 1) if the current CF input level is above or below its average, decrease or increase the tonic firing rate of PC, respectively; (spike generator rule 2) if the current CF input level is close to its average, then return the tonic firing rate of the PC to its pre-lesion steady state level (cf. Eq. 27). The first rule is consistent with the observation of an increment and decrement of simple spike activity of PCs after inactivation of IO[3] and repetitive stimulation of CFs[3,4], respectively. The second rule is consistent with experimental data showing that PCs recover their pre-lesion level baseline firing rate months[5] or years[6] after an IO lesion.

The model explains the influence of an IO lesion on PC firing rate as follows. First, when the IO is damaged, PCs do not get a CF input and thus become hyperactive (spike generator rule 1; see the initial rising red curve in Figure S3A). This hyperactivity declines with time (the slow falling red curve in Figure S3A) to its pre-lesion level as the temporal average of CF activity slowly approaches its current value, i.e., zero (spike generator rule 2).

The model shows that the strange kinetics of post-lesion DCN activity could be attributed to internal processes in DCN neurons. Figure S3B shows four variables that are relevant to the behavior of the DCN neuron. The brown and purple curves in Figure S3B are the weighted input from the PC population and its temporal average, respectively. The model hypothesizes that the PC population discharge modulates the DCN activity with at least three different temporal scales. The first modulation component has a slow temporal scale, thus it represents an absolute level of the inhibitory input. The second component has an intermediate temporal scale that measures the level of inhibitory input compared to an average inhibitory input from the PC population (current input from PC population – intermediate temporal average of the input from PC population) with a time constant ~2.7 months (see [5]), which represents the observed slow structural remodeling process at the PC axon terminals[6]. The average PC population input is shown in a purple curve in Figure S3B. The second modulatory component (brown curve minus purple curve) is shown in the green curve in Figure S3B. The third modulatory component has a short temporal scale that compares the fast scale difference in PC population activity with a time constant of ~100 msec (see [7]). This fast scale component is responsible for the known rebound discharge in DCN neurons (McKay et al. 2005). Because this fast scale component has significantly faster kinetics compared to the relatively slow kinetics of the PC in response to the IO lesion (time constant ~20 sec; see [8]) the fast scale modulatory component does not play an important role in the post-lesion DCN activity. Therefore, the activity of DCN neurons is controlled by the two components of slow and medium scale modulatory mechanisms (brown and green curves). Note that the DCN activity (blue curve in Figure S3A) has a similar profile to that of the medium scale component (green curve in Figure S3B).

The model explains the large post-depression rebound peak (~200% of pre-lesion level) and the subsequent damped oscillatory behavior of DCN using an interaction among three variables of current PC input (brown curve in Figure S3B), its average (purple curve in Figure S3B) and the synaptic efficacy at the PC🡪DCN synapse (black curve in Figure S3B). The efficacy of the simulated synapse at PC🡪DCN obeys the synaptic rules as in Eq. (52). Using those rules the damped oscillatory behavior of DCN, which resembles that of the medium scale component (green curve in Figure S3B), can be explained as follows. First, the quick increment of PC population input relative to its average (green curve = brown curve – purple curve in Figure S3B) pushes the intermediate variable below zero. Second, as the PC population activity recovers to its pre-lesion level, the once increased synaptic efficacy due to the increased PC activity (the first multiplicative term in Eq. (52)) returns to its pre-lesion level (the second multiplicative term in Eq. (52)). This decrement of synaptic efficacy makes the level of the weighted input (brown curve) decrease further, passing below its average (purple curve) because of the faster kinetics of the brown curve compared to the purple curve. Also, this smaller-than-average PC input further facilitates the depression of the synaptic efficacy. As this decrement of weighted input continues, the depression of the synaptic efficacy slows down due to its lower bound, thus giving the average weighted input variable (purple curve) some time to catch up to the PC input (brown curve). As the two variables come close once again it triggers the upswing of the synaptic efficacy variable (the second multiplicative term in Eq. (52)). This interaction between the three variables continues, generating an oscillation in the green curve that reflects the difference between the brown curve and the purple curve. The oscillation, however, decays away because of the slow overall decrement of the synaptic efficacy at PC🡪DCN synapse. This occurs because of the absence of an IO input to DCN, which makes the synaptic efficacy decrease (the first term in Eq. (52)). Thus, whereas the medium scale component of the PC modulation generates the damped oscillation, the overall activity of the DCN is determined by the contributions of both the large and medium scale modulatory components. The mathematical descriptions of the lumped model are given in the following.

***Purkinje cell:*** The lumped PC has one dendrite that gets lumped inputs from PF and IN. Before the IO lesion, the baseline CF signal of 2.6x10^-3^ (~1.5 Hz), which comes from the average of the original simulation, was provided to the PC. For the simulation of the IO lesion, the CF signal was set to zero for the duration of the simulation. The membrane potential of the dendrite (*PCBr*) obeys the following rule:

$\frac{dPCBr}{dt}=-PCBr+Input+\beta\left(PC-PCBr\right)\text{ , }$ (S1)

where the first term of the right side of the equation is a leakage term; *Input* represents the lumped input from INs and PF, and is set to 0.205; the third term is a conduction term between the soma and the dendrite with the conductance *β* being 1.5. The lumped value of *Input* was chosen to reflect the inputs of the original model. Also, the static potential of PCBr was checked to verify the equivalence between the original and lumped versions (see Figure S3).

The soma of the lumped model PC communicates with its dendrite and its membrane potential as follows:

$\frac{dPC}{dt}=-PC+T\left(1-PC\right)\text{ }+2\beta\left(PCBr-PC\right)\text{, }$ (S2)

where the first term is the leakage component; the second term represents the tonic component of the cell; the last part describes the potential due to the current between the soma and the attached dendrites. 2*β* is the conductivity between the dendrite and the soma and the multiplier (2) reflects the two dendrites of the original version. The tonic component *T* that influences the PC’s baseline firing rate is the same as in Eq. (27).

***Deep cerebellar nucleus neuron:*** The lumped model of deep cerebellar nucleus has one excitatory neuron (DCN). The inhibitory neuron (IDCN) is not needed because its target, IO, no longer exists due to the lesion. Before the lesion of IO, the baseline input from IO of 2.6x10^-4^ (~1.5 Hz), which comes from the average of the original simulation, was provided to the DCN. For the simulation of an IO lesion, the IO signal value of zero was provided for the duration of the simulation. The simulated DCN neuron receives one excitatory input from MFs and inhibitory input from the lumped PC. The membrane potential of DCN neuron is simulated as follows:

$\begin{matrix} \frac{dDCN}{dt}=-aDCN\text{ }+\left\{b\left[M\right]^{+}I_{MF}+c\left(\left[M\right]^{+}+(h_{DCN}-DCN)\left[-M\right]^{+}\right)\right\} \\ \text{ }+T_{DCN}-d\cdot I_{PC}\text{, } \end{matrix}$ (S3)

where the value of MF input, *I_MF_* (0.036), is the average value of the MF input in the original model; the input from PCs is denoted as *I_PC_* and defined as

$I_{PC}=54w_{PCDCN}PC.\text{ }$ (S4)

where 54 is the number of PCs in the original simulation. All the rest of the equations are the same as the ones given in the main text.

***Note 4. Variables in the model***

To facilitate understanding of the model, a cartoon with variables in corresponding anatomical modules of the network is provided in Supplementary Figure S4.

**References**

1. Lampl I, Yarom Y (1993) Subthreshold oscillations of the membrane potential: a functional synchronizing and timing device. J Neurophysiol 70: 2181-2186.

2. Lang EJ, Sugihara I, Welsh JP, Llinas R (1999) Patterns of spontaneous purkinje cell complex spike activity in the awake rat. J Neurosci 19: 2728-2739.

3. Colin F, Manil J, Desclin JC (1980) The olivocerebellar system. I. Delayed and slow inhibitory effects: an overlooked salient feature of cerebellar climbing fibers. Brain Res 187: 3-27.

4. Demer JL, Echelman DA, Robinson DA (1985) Effects of electrical stimulation and reversible lesions of the olivocerebellar pathway on Purkinje cell activity in the flocculus of the cat. Brain Res 346: 22-31.

5. Batini C, Billard J, Daniel H (1985) Long term modification of cerebellar inhibition after inferior olive degeneration. Exp Brain Res 59: 404-409.

6. Billard JM, Daniel H (1988) Persistent reduction of Purkinje cell inhibition on neurones of the cerebellar nuclei after climbing fibre deafferentation. Neurosci Lett 88: 21-26.

7. McKay BE, Molineux ML, Mehaffey WH, Turner RW (2005) Kv1 K+ channels control Purkinje cell output to facilitate postsynaptic rebound discharge in deep cerebellar neurons. J Neurosci 25: 1481-1492.

8. Cerminara NL, Rawson JA (2004) Evidence that climbing fibers control an intrinsic spike generator in cerebellar Purkinje cells. J Neurosci 24: 4510-4517.

**Figure Legends**

Figure S1. Conditioned responses of the DCN with (A) or without (B) short-time scale component (LTP/LTD). Panels A and B show that LTP/LTD do not contribute to the conditioned eye blink response. Some differences in the traces between the two figures are due to the initial small random variations in the parameters of the IO neurons used to simulate the natural firing frequency differences between neurons. This small randomness in turn generates some simulation-to-simulation differences in Purkinje and DCN activities.

Figure S2. Normal behavior of model. A. Firing activity of IO neurons and the effect of excitatory input. The point when an external input is given is indicated by an arrow. The neurons quasi-randomly fire with patch synchronization (indicated by circles) when there is no external excitatory input. When an external input is given, the neurons fire synchronously (see the peak in spike histogram at the bottom). Note that the stimulus-induced synchronized firing does not happen for every IO neuron. B. Example of stimulus-induced firing. This example shows spiking by an IO neuron when an excitatory input is given. Note the delay of tens of msec. C. Example of failed firing at external input. This example shows that IO firing is highly dependent on the internal state of individual neurons. The external stimulus just resets the subthreshold oscillation (see the arrow) without driving it enough to spike. D. PC weighted input to DCN neuron. Note that even with the synchronous discharges of IO neurons the output of PC population does not change significantly (arrow). E. Normal state DCN activity. When there is no input to the IO, the firing activity of IO neurons is asynchronous, giving background noisy input to DCN. Even when there is some level of IO synchrony in firing, its impact on DCN discharge is limited (arrow). This fact becomes clear when the firing rate of a DCN neuron during a classically conditioned response is considered (F). F. Learned DCN activity during a CR phase in classical delay conditioning. Comparing the CR-related amplitude with the amplitude caused by the moderately synchronized IO activity makes it clear that ordinary movements require relatively large output from the DCN. Note that the large CR-related amplitude of the DCN activity has been learned by the cerebellar network with the same kind of synchronized IO activity as the one shown in (A).

Figure S3. The correspondence of the lumped model to the original model (A) and internal variables (B). A. The lumped model’s behavior (PC: red, DCN: blue) matches to that of the original model (PC: thick orange, DCN: thick cyan) over one day of simulation. B. DCN neuron’s internal variables that shape the DCN firing behavior. Black curve: rescaled (x10) synaptic efficacy at PC🡪DCN. Brown curve: weighted input from the PC population to DCN neuron. Purple curve: temporal average of weighted PC population input. Green curve: the medium-scale modulatory component (brown curve – purple curve).

Figure S4. Variables in corresponding anatomical modules in the model are shown. The numbers in the parentheses correspond to the equation numbers in the text. *M* in the DCN indicates the modulation by the PCs.
