# Supplementary figures and images for: Interaction between Purkinje Cells and Inhibitory Interneurons May Create Adjustable Output Waveforms to Generate Timed Cerebellar Output
Source: PLoS One. 2008 Jul 23;3(7):e2770. doi: 10.1371/journal.pone.0002770 (PMC2474676; doi:10.1371/journal.pone.0002770)

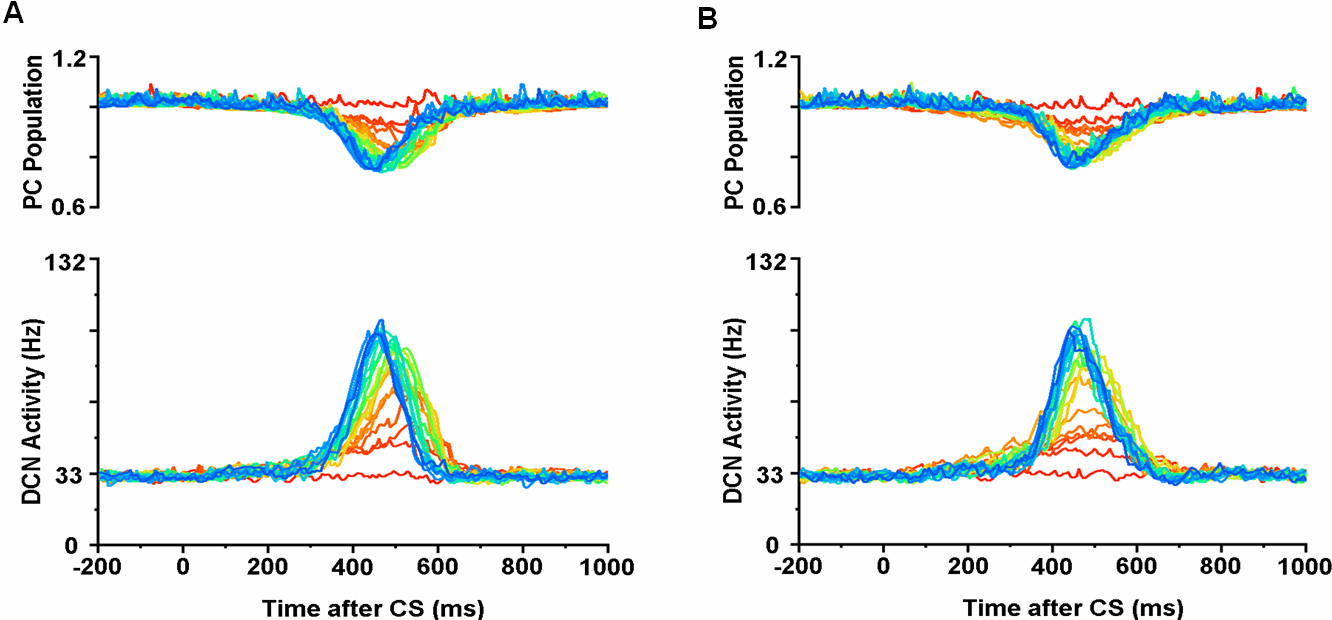

Supplement: Figure S1 — Conditioned responses of the DCN with (A) or without (B) short-time scale component (LTP/LTD). Panels A and B show that LTP/LTD do not contribute to the conditioned eye blink response. Some differences in the traces between the two figures are due to the initial small random variations in the parameters of the IO neurons used to simulate the natural firing frequency differences between neurons. This small randomness in turn generates some simulation-to-simulation differences in Purkinje and DCN activities. (0.28 MB TIF) [file pone.0002770.s002.tif]

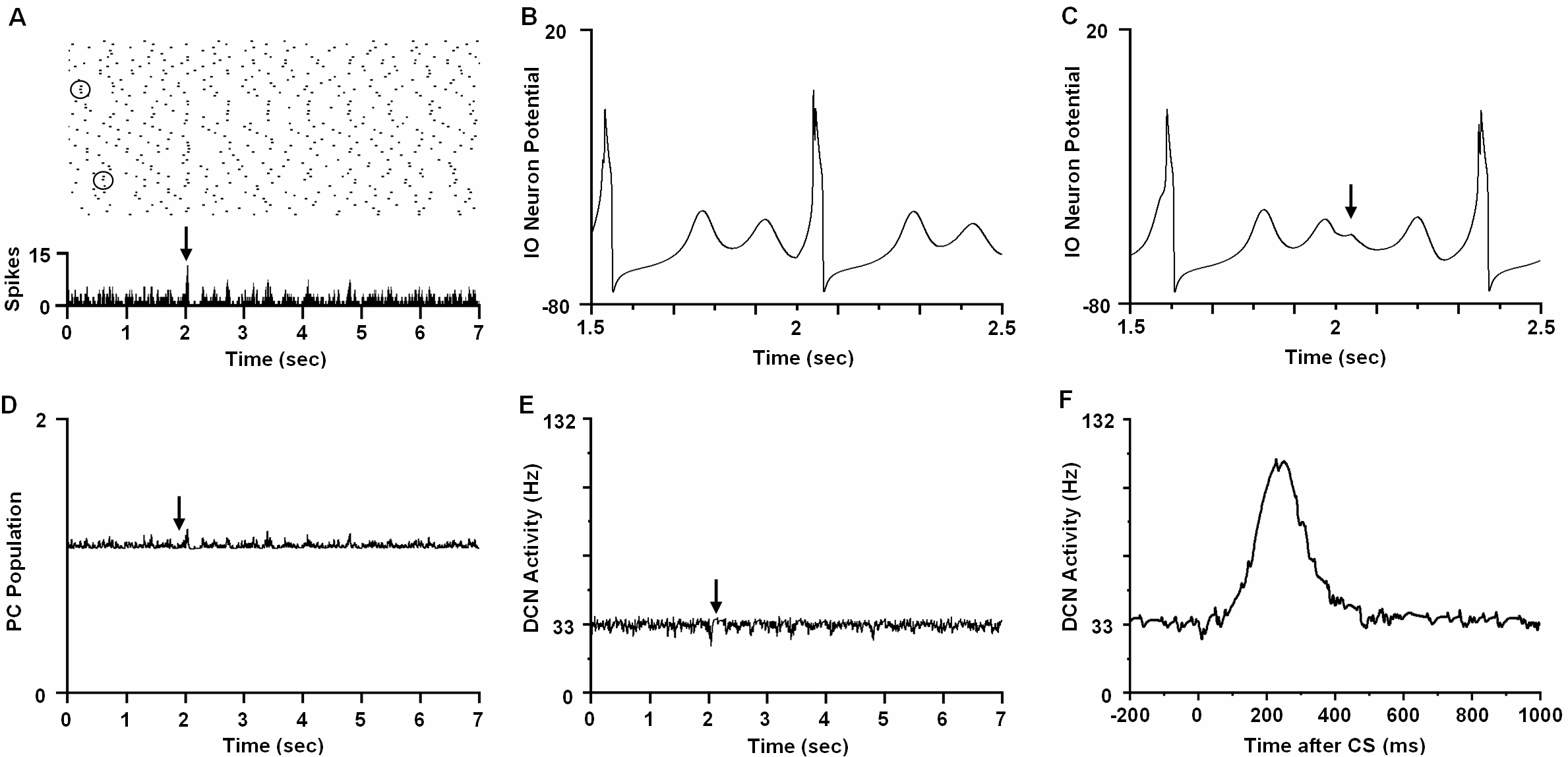

Supplement: Figure S2 — Normal behavior of model. A. Firing activity of IO neurons and the effect of excitatory input. The point when an external input is given is indicated by an arrow. The neurons quasi-randomly fire with patch synchronization (indicated by circles) when there is no external excitatory input. When an external input is given, the neurons fire synchronously (see the peak in spike histogram at the bottom). Note that the stimulus-induced synchronized firing does not happen for every IO neuron. B. Example of stimulus-induced firing. This example shows spiking by an IO neuron when an excitatory input is given. Note the delay of tens of msec. C. Example of failed firing at external input. This example shows that IO firing is highly dependent on the internal state of individual neurons. The external stimulus just resets the subthreshold oscillation (see the arrow) without driving it enough to spike. D. PC weighted input to DCN neuron. Note that even with the synchronous discharges of IO neurons the output of PC population does not change significantly (arrow). E. Normal state DCN activity. When there is no input to the IO, the firing activity of IO neurons is asynchronous, giving background noisy input to DCN. Even when there is some level of IO synchrony in firing, its impact on DCN discharge is limited (arrow). This fact becomes clear when the firing rate of a DCN neuron during a classically conditioned response is considered (F). F. Learned DCN activity during a CR phase in classical delay conditioning. Comparing the CR-related amplitude with the amplitude caused by the moderately synchronized IO activity makes it clear that ordinary movements require relatively large output from the DCN. Note that the large CR-related amplitude of the DCN activity has been learned by the cerebellar network with the same kind of synchronized IO activity as the one shown in (A). (0.32 MB TIF) [file pone.0002770.s003.tif]

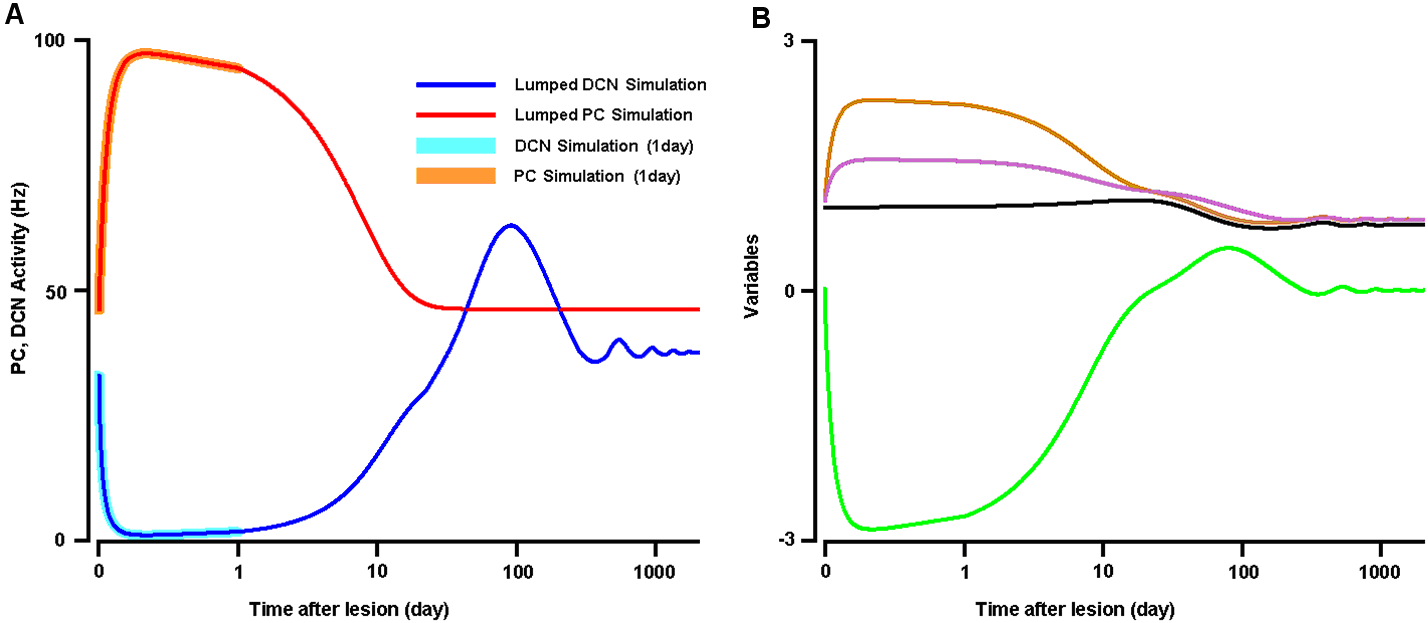

Supplement: Figure S3 — The correspondence of the lumped model to the original model (A) and internal variables (B). A. The lumped model's behavior (PC: red, DCN: blue) matches to that of the original model (PC: thick orange, DCN: thick cyan) over one day of simulation. B. DCN neuron's internal variables that shape the DCN firing behavior. Black curve: rescaled (×10) synaptic efficacy at PC⇒DCN. Brown curve: weighted input from the PC population to DCN neuron. Purple curve: temporal average of weighted PC population input. Green curve: the medium-scale modulatory component (brown curve - purple curve). (0.11 MB TIF) [file pone.0002770.s004.tif]

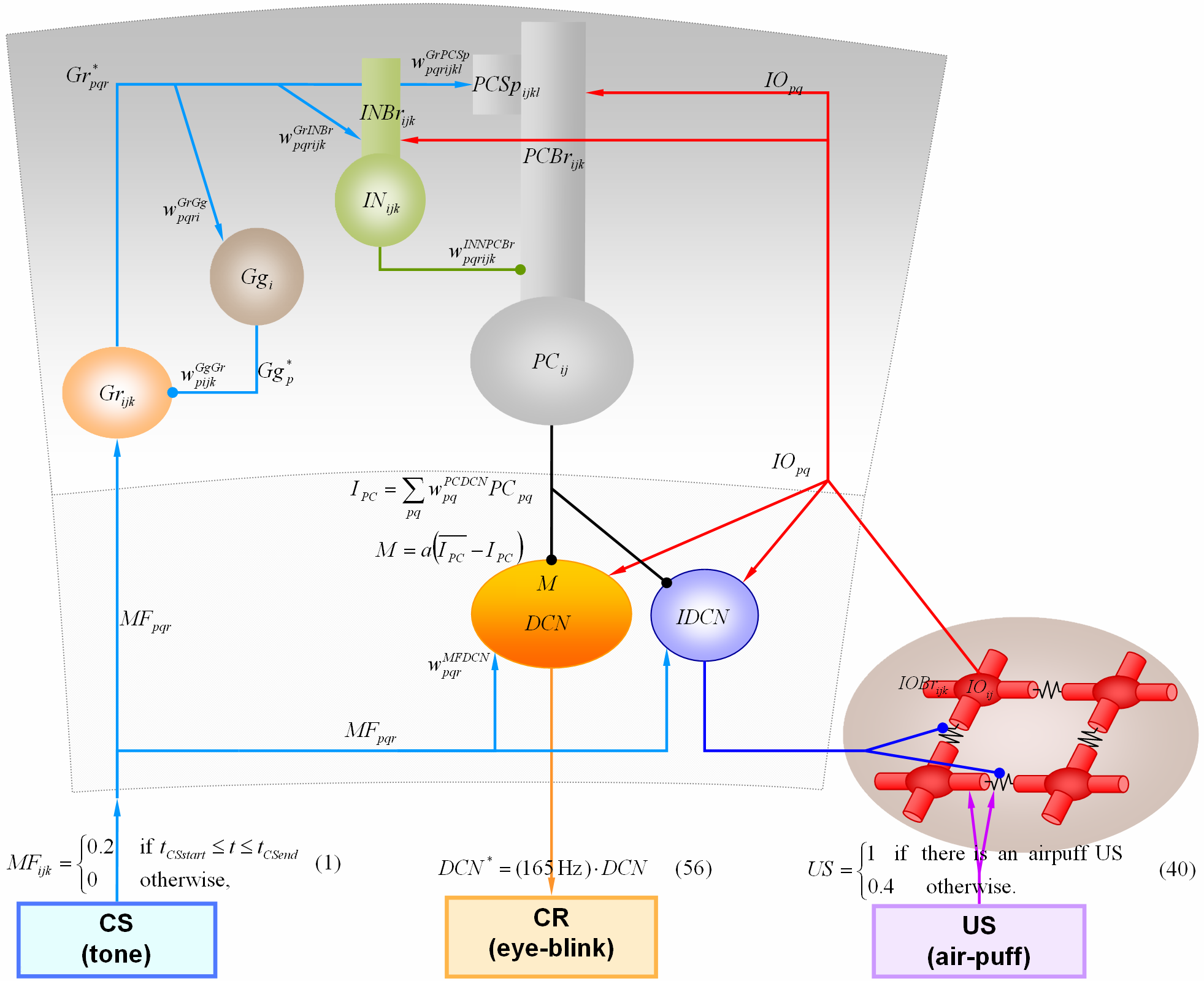

Supplement: Figure S4 — Variables in corresponding anatomical modules in the model are shown. The numbers in the parentheses correspond to the equation numbers in the text. M in the DCN indicates the modulation by the PCs. (0.95 MB TIF) [file pone.0002770.s005.tif]
